# Wearable Sensor Systems for Fall Risk Assessment: A Review

**DOI:** 10.3389/fdgth.2022.921506

**Published:** 2022-07-14

**Authors:** Sophini Subramaniam, Abu Ilius Faisal, M. Jamal Deen

**Affiliations:** ^1^School of Biomedical Engineering, McMaster University, Hamilton, ON, Canada; ^2^Electrical and Computer Engineering, McMaster University, Hamilton, ON, Canada

**Keywords:** fall risk assessment, fall detection, wearables, smart insole, inertial sensors, plantar pressure, gait analysis, machine learning

## Abstract

Fall risk assessment and fall detection are crucial for the prevention of adverse and long-term health outcomes. Wearable sensor systems have been used to assess fall risk and detect falls while providing additional meaningful information regarding gait characteristics. Commonly used wearable systems for this purpose are inertial measurement units (IMUs), which acquire data from accelerometers and gyroscopes. IMUs can be placed at various locations on the body to acquire motion data that can be further analyzed and interpreted. Insole-based devices are wearable systems that were also developed for fall risk assessment and fall detection. Insole-based systems are placed beneath the sole of the foot and typically obtain plantar pressure distribution data. Fall-related parameters have been investigated using inertial sensor-based and insole-based devices include, but are not limited to, center of pressure trajectory, postural stability, plantar pressure distribution and gait characteristics such as cadence, step length, single/double support ratio and stance/swing phase duration. The acquired data from inertial and insole-based systems can undergo various analysis techniques to provide meaningful information regarding an individual's fall risk or fall status. By assessing the merits and limitations of existing systems, future wearable sensors can be improved to allow for more accurate and convenient fall risk assessment. This article reviews inertial sensor-based and insole-based wearable devices that were developed for applications related to falls. This review identifies key points including spatiotemporal parameters, biomechanical gait parameters, physical activities and data analysis methods pertaining to recently developed systems, current challenges, and future perspectives.

## Introduction

Falling can lead to adverse health outcomes, especially in older adults. Detecting falls as they occur, and more importantly, preventing falls through the assessment of fall risk can prevent detrimental health effects, which may otherwise occur with falling. Fall risk assessment and fall detection have been accomplished through diverse methods, with a growing area being in wearable technologies (1). Other methods to assess fall risk and detect falls include through smart home technologies (2–4), camera-based systems (5), smartphone sensors (6) as well as other non-contact and wireless methods (7, 8), such as Monopulse Doppler radar used for the detection of falls of multiple individuals (9).

Wearable devices are ideal for monitoring health due to the small size of low-cost, user-friendly devices that can allow for continuous activity monitoring and physiological data acquisition during daily activities. The limitations of traditional laboratory-based activity monitoring systems, such as monitoring in unfamiliar settings, large-scale set-up or impacts on natural gait when being aware of monitoring, are also overcome with wearable systems (10). Commonly used wearable systems for this purpose are inertial measurement units (IMUs) which acquire data from accelerometers and gyroscopes. IMUs can be placed at various locations on the body to acquire motion data that can be further analyzed, combined and interpreted. Insole-based devices are wearable systems that were also developed for fall risk assessment and fall detection. The insole-based systems are placed beneath the sole of the foot and typically obtain plantar pressure distribution data.

Various portable monitoring systems exist for healthcare, which include wireless non-wearable systems, phone-based non-wearable systems, non-integrated wearable systems and fully integrated wearable systems (11, 12). Non-integrated systems typically allow for comprehensive and continuous monitoring, but are usually not comfortable for the user, are expensive and not ideal for long-term use. On the other hand, fully integrated wearable systems are smaller, more affordable and are a better choice for long-term use (12). Inertial sensor systems and insole-based sensor systems are typically fully integrated, making them ideal portable monitoring systems to be used for fall risk assessment over long periods of time. Wearable technologies typically analyze gait characteristics using various sensors including pressure sensors and inertial sensors. These devices analyze gait characteristics and their variability for fall risk assessment, fall detection and slip detection. Some examples of gait characteristics analyzed include stride length, stance/swing phase ratio, plantar pressure distribution and center of pressure trajectory. Once data is acquired from these sensor systems, data processing, data fusion, and various analysis techniques such as machine learning models or manual data analysis, are employed to obtain meaningful information from the parameters investigated (10, 13).

Insole-based devices typically incorporate pressure sensors to measure plantar pressure or plantar pressure distribution (PPD) from the plantar aspect of the foot. Inertial sensors used for gait analysis are typically accelerometers and gyroscopes placed on the leg (such as on the foot, shank or thigh), lower back or hip. Insole-based pressure data has also been analyzed alongside inertial sensors placed at the lower leg or plantar aspect of the foot to obtain additional information regarding an individual's gait characteristics.

**Figure 1:**
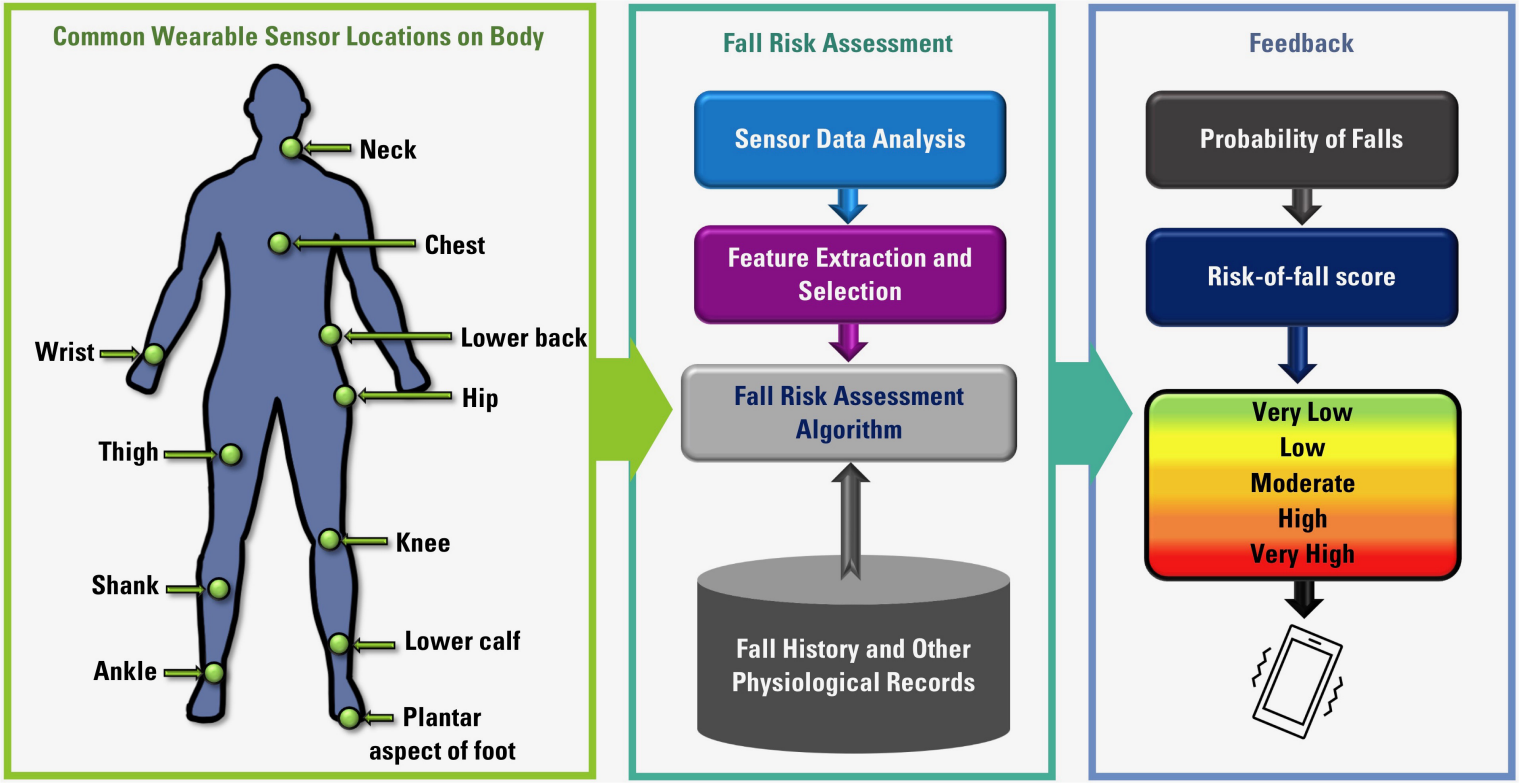
Overall flow diagram of fall risk assessment using wearable sensors.

Other wearable sensors have been used to assess fall risk and detect falls beyond pressure sensors and inertial sensors, including but not limited to photoplethysmography (PPG) sensors, electromyography (EMG) sensors and galvanic skin response (GSR) sensors. In this article, we discuss various wearable systems used to investigate parameters relevant to fall risk assessment and fall detection, with a focus on inertial and insole-based systems. The overall flow diagram of fall risk assessment using wearable systems is presented in Figure 1.

In recent years, there have been advancements in diverse wearable technologies. Among these, several inertial-based systems, insole-based sensor systems, and other wearable technologies have been developed for the purpose of gait analysis, plantar pressure distribution determination and for fall risk mitigation (14–16).

Wearable technologies that have been recently developed for the assessment of fall risk and fall detection using insole-based or inertial sensor-based systems are summarized in this article. Here, an overview is provided introduction (section Introduction), and the article search method is described in section Search Method. Background information regarding falling is provided in section Overview of Falling. In section Inertial-Based Sensor Systems and section Insole-Based Sensor Systems, information regarding relevant inertial-based sensor systems and insole-based systems, respectively, are provided. These sections include background information regarding the sensor systems used and a review of existing research systems used for fall risk assessment and fall detection. Wearable sensor systems other than inertial-based and insole-based technologies are concisely discussed in section Other Wearable Systems, which is followed by information regarding Fall Risk Assessment and Modeling Techniques provided in section Fall Risk Assessment and Modeling Techniques. In section Challenges, the challenges associated with existing wearable technologies for fall risk assessment are outlined, with suggestions for future research presented in section Future Research Perspectives. Conclusions are lastly presented in section Conclusions.

## Search Method

This article is a narrative review which employed a systematic search process. Electronic database searches were performed in PubMed and Web of Science databases, with final searches completed in March 2022. The databases were searched using Boolean operators and focused keywords relevant to fall risk and wearable devices, resulting in 305 articles. The search strategy used a combination of terms related to falls and fall risk, wearables, and the sensor location {[(“Fall risk”) OR (Fall) OR (Faller^*^) or (Falls)] AND [(“Wearable sensors”) OR (“Wearable devices”) OR (Wearable^*^)] AND [(Foot) OR (Feet) OR (“Lower-leg”) OR (“Lower leg”) OR (Leg) OR (Body) OR (”Whole body”) OR (“Full body”)]}. This search was following by a title and abstract screening process using the Covidence software, which led to 150 articles being selected for review. Upon full-text examination of relevant articles, 21 selected articles are presented in this work, with an emphasis on insole-based and inertial-based wearable devices for fall risk assessment and fall detection. Only studies reporting on fall risk assessment and fall detection using insole-based or inertial sensors were included. Studies published before the year 2000 were excluded. Searches and screening of articles were conducted by authors of this review article.

## Overview of Falling

### The Relationship Between Fall Risk, Fear of Falling and Falls

**Figure 2:**
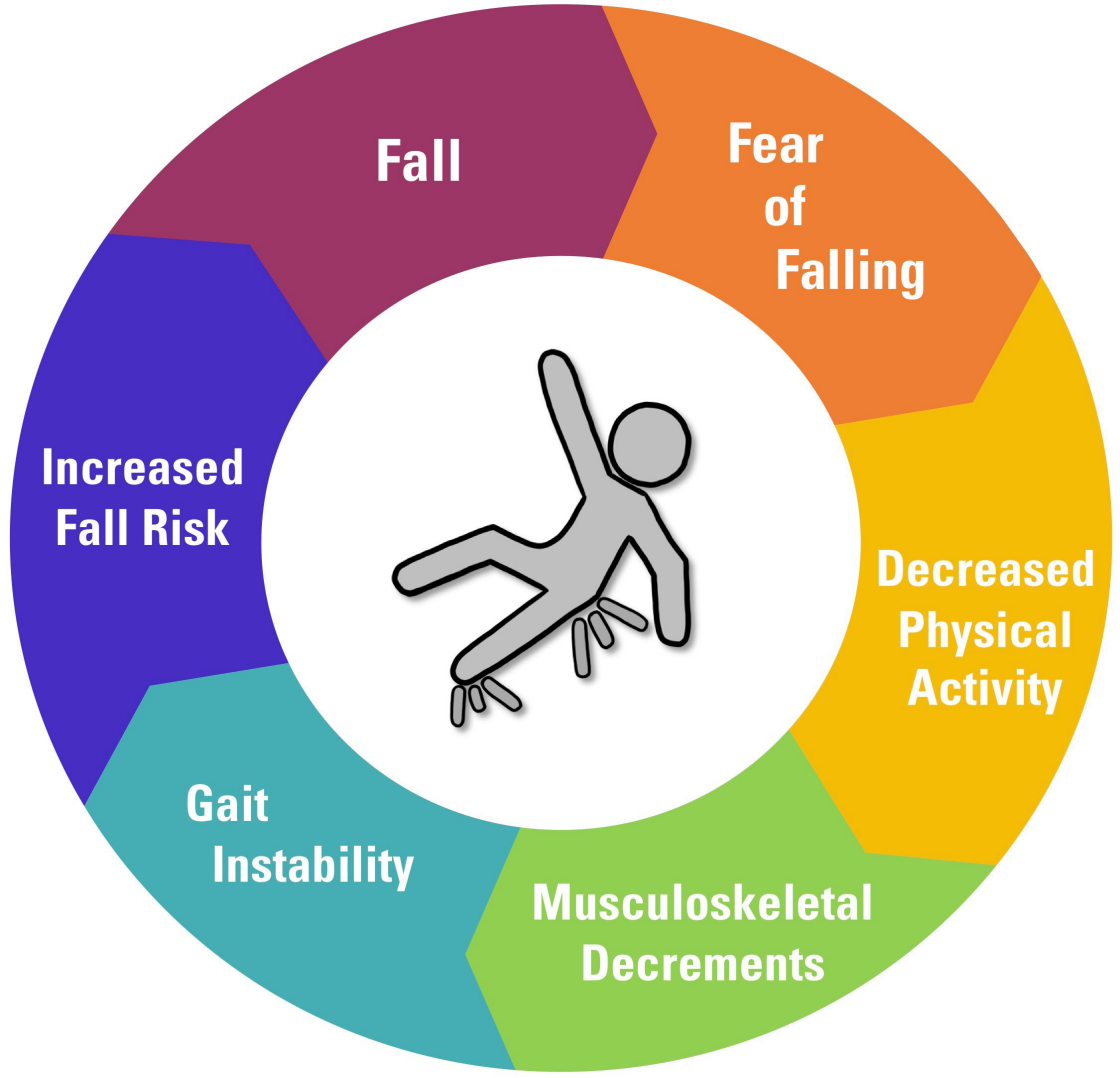
The cycle of falling.

Falling is very common among members of the aging population (17), so identifying an individual's risk of falling in advance can lead to the prevention of falls and better health outcomes. Beyond the detrimental effects of falling, the *fear of falling* can also lead to adverse health outcomes. In the case of older adults, having experienced a fall once can lead to an increased fear of falling that impacts daily life activities (18). This is of particular concern as fear of falling can cause individuals to not engage in healthy and beneficial activities, such as physical exercise activities that can otherwise provide great benefits to health. This decrease in physical activity due to an assumed risk of falling can further increase an individual's fall risk as certain factors such as cognitive ability or muscle strength may decline without an active lifestyle (19, 20). This decrease in activity contributes to an increased fall risk, leading to a further decrease in activity in a positive feedback cycle (Figure 2). According to Statistics Canada, 20% of older adults overestimated their fall risk in 2009 (19). Correctly identifying contributing factors and the extent to which such factors can affect fall risk can allow for the prevention of unnecessary fears of falling and lead to healthier lifestyles.

### Factors Contributing to Fall Risk

Several factors can contribute to one's risk of falling. These include physiological, psychological, behavioral and environmental factors. Factors contributing to fall risk may be intrinsic or extrinsic (21). Intrinsic factors refer to those that concern the individual, whereas extrinsic factors refer to the environment. These factors may independently contribute to increasing one's risk of falling or act synergistically.

There exist various methods to assess fall risk that aim to quantify certain contributors. For example, the Fall Risk Assessment Checklist for Home Health Care by John Hopkins Medicine scores patients based on factors including age, fall history, medication use, tethered patient care devices, mobility/gait considerations and cognitive considerations (22). Based on the total number of points, the patient may be classified as having “moderate fall risk” or “high fall risk.” Various assessment methods and tools use different factors to assess fall risk, which may be specific to the target population of the assessment, environment, age, or specific medical conditions. Similarly, wearable sensor systems monitor and analyze gait characteristics to determine an individual's risk of falling. Preventing or addressing the factors that can contribute to falling can help reduce fall risk and allow individuals to lead a healthier lifestyle.

### Importance of Fall Prevention

Preventing falls is crucial as falling may otherwise lead to adverse health outcomes. In fact, among older adults in Canada, falls are the leading cause of injury (23). Older adults have a higher risk for more severe adverse outcomes after a fall. These include an increased fatality risk, decreased quality of life, acute and chronic pain, hospitalizations, and long-term changes to an individual's lifestyle (19). Thirty three percentage of Canadian older adults that have been hospitalized after a fall end up living in long-term care homes, which is an example of a single fall leading to severe adverse outcomes, which highlights the need to appropriately assess fall risk.

### Assessing Fall Risk

Fall risk assessment tools can be extremely useful in preventing falls. An individual's fall risk can be assessed by several methods, including assessment checklists, in laboratory settings, through smart-home systems or using wearable sensors (21, 24). Wearable technologies are a major and very useful area of research regarding fall risk assessment and fall detection. Wearable devices are particularly of use as measurements can be taken in a user's natural setting, during daily activities and in a convenient manner. In the following sections, we describe recent wearable technologies that have been developed and used toward fall risk assessment.

## Inertial-Based Sensor Systems

### Overview

**Figure 3:**
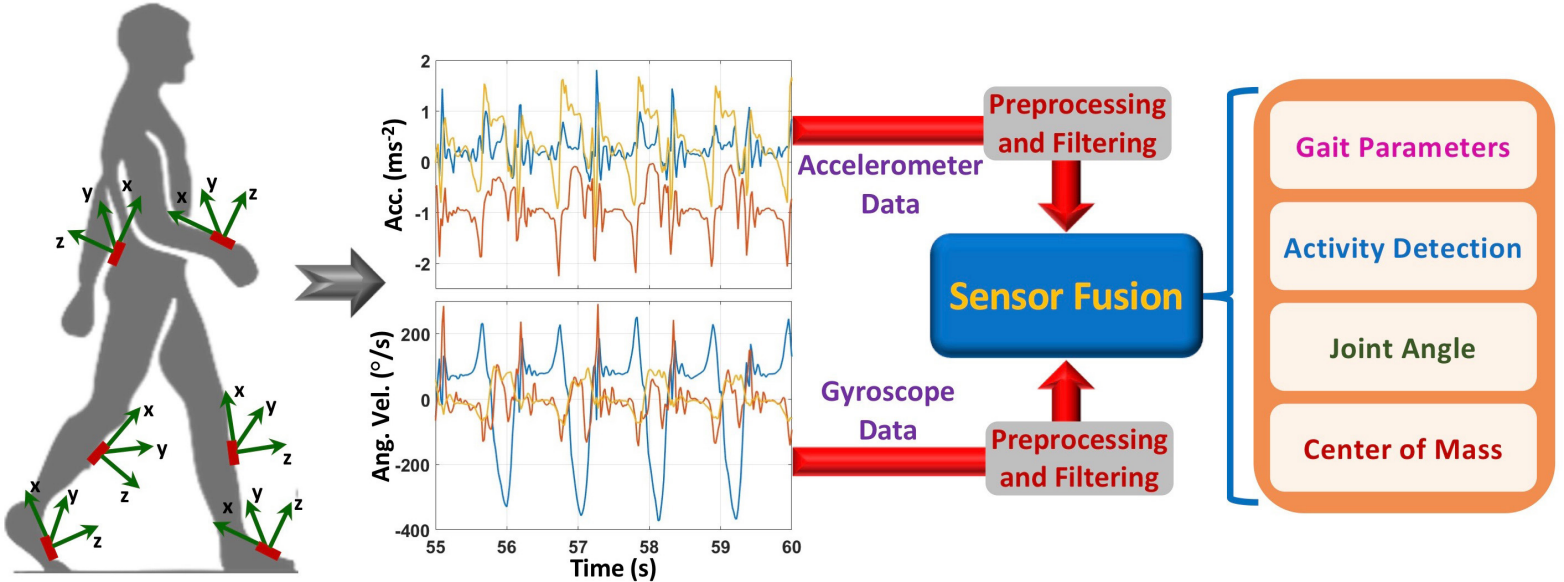
Activity and gait monitoring for fall risk assessment and fall detection using inertial sensors.

Inertial sensors are the most widely used wearable sensors for fall risk assessment and fall detection. An inertial sensor, also known as an inertial measurement unit–IMU, comprises of multiple 3-axis sensors (mainly accelerometers and gyroscopes) to measure linear acceleration and angular velocity in their own three-dimensional local coordinate system. Moreover, compared to other sensor technologies, inertial sensors are a very promising sensor unit for developing a wearable monitoring system, because of their low cost, compact size and capability to measure motion-related parameters with high precision and accuracy (25–30). Therefore, they are suitable to monitor body movement and motion. Normally, inertial sensors are attached to different body parts to measure body motion-related parameters such as acceleration and speed of movement, joint angle, and rotation during movement (Figure 3).

Recently, researchers are intensely investigating the potential of instrumented fall-risk assessment and prediction tools and inertial sensors seem to be the first choice of many researchers (31–40). To collect the data, the aforementioned studies used between one to five inertial sensors attached to the human body. Most of the studies used data from the body-attached sensors to distinguish different fall status groups or to classify falls during different fall-risk assessment tasks such as walking, quiet standing, single-task, dual-task, one leg standing, sit-to-stand transitions, and from timed up and go (TUG) test (41). There are three important factors to consider when designing an effective inertial sensors-based fall-risk assessment system: placement of the sensor, task to be performed, and key features to be extracted and analyzed.

### Placement of the Inertial Sensors

Based on this survey, it was observed that most of the studies placed the sensors close to the center of the body (e.g., lower back, waist) to estimate body orientation (tilt), posture, and postural transition duration between different activities as these features are highly associated with fall risk (42). When an individual has an abnormal body orientation during any activity, it can lead to a fall. An inertial motion sensor attached near to the body center can detect the body orientation and assess the risk of a fall. The sensor in this location can also estimate the duration of a posture as well as transition time between two postures. Balance and stability of the body during postural transitions are very important and recognized as the key factors for assessing falls. Higher transition duration indicates lower balance and stability and consequently higher fall risk. The duration can be estimated using the acceleration (from the movement of the body) and angular rotation (inclination of the trunk) signals from the inertial sensors. Other proposed sensor locations were foot, leg, wrist, chest, and head to measure additional key parameters such as acceleration and velocity during locomotion, foot state as well as gait stability and symmetry (43, 44). Using multiple locations allow the system to estimate total body posture, coordination between different limbs as well as to perform complete functional assessment to predict the fall risk.

### Fall Risk Assessment Tests

There are several well-established fall risk assessment tests to estimate future fall probabilities. These tests usually involve a set of questionnaires and functional assessments gait, posture, cognition, and other fall risk factors. Although these tests are subjective and qualitative, incorporating inertial sensors during these tasks can provide a quantitative assessment of fall risk as well as classify people as fallers and non-fallers. This combination will also allow us to evaluate balance during locomotion and lower limb strength. The most common fall risk assessment tests are timed up and go (TUG) (45), Berg Balance Scale (BBS) (46), sit to stand (STS) (47), and one leg stand (OLS) (48).

### Extracted Features

Features from the inertial sensors can be divided into several categories such as spatial, temporal, frequency, linear acceleration, angular, and non-linear features. Linear acceleration features are related to postural stability during activities. Spatial features include step length, stride length, step width and number of steps. that can be estimated from inertial signals measured during the walking. On the other hand, temporal features included time related features such as cadence, step time, stride time, gait speed, stance and swing time, single and double support time, time during only one foot and both feet are in contact with the ground, gait cycle, and coefficient of variation (CV) for step. These spatial and temporal features are widely used in gait analysis (49). The most common frequency features extracted from the inertial sensors are the harmonic ratios (HRs) of acceleration in different directions (mediolateral, anteroposterior, and longitudinal axis) (33, 34). The frequency features are often used to estimate the stability and smoothness of body movement during gait (50). The angular features include joint range of motion, and rotation during gait and movements. In addition to those features, some non-linear features related to gait dynamic stability (e.g., the maximum Lyaponuv exponent, multiscale entropy, and recurrence quantification analysis) are reported in (51, 52) that have the association with fall history. As different features represent different gait and movement characteristics, an appropriate feature combination is required to employ a machine learning technique to distinguish fallers and non-fallers as well as to compare features between groups of fallers and non-fallers. Therefore, considering the heterogeneity of the features is important to consider when developing an effective fall risk assessment model. However, there is still a lack of consensus about the features that are optimal for fall studies, especially for different subject groups (49). Moreover, there can be significant differences when the analysis is performed in a retrospective or a prospective way (33).

In order to develop an efficient fall risk assessment tool, appropriate features should be selected with a combination of proper sensor placement and fall risk assessment test. Different optimal combinations of these factors are proposed in different studies that are summarized below in next section as well as in Table 1.

**Table 1:** Studies using inertial sensor-based fall-risk assessment and fall detection systems.

**Reference**	**Participants**	**Sensor system (Device location)**	**Parameters investigated**	**Analysis techniques**	**Key points**
Bautmans et al. (31)	121 • 40 older adults with increased fall-risk (80.6 ± 5.4 years) • 41 old controls (79.1 ± 4.9 years) • 40 young controls (21.6 ± 1.4 years)	• 1 3-D piezoresistive accelerometer (sacrum - between the spinae ilaca posterior superior)	• Gait parameters: gait speed, step-time asymmetry, mediolateral and craniocaudal step and stride regularity • Functional parameters: cognition (MMSE), dependency, grip strength, muscle endurance, and fall-risk (fall-history, timed-get-up-and-go and Tinetti-test)	• Analysis of variance (ANOVA) • Logistic regression	• Only gait speed presented discriminative result for increased fall-risk • Gait variability features showed good relationships (*p* < 0.05) with functional characteristics for older adults
Kumar et al. (32)	–	• 1 pressure sensor (arm) • 1 Infra-red sensor (fingertip) • 1 3D accelerometer (waist)	• Blood pressure • Heart rate • Blood glucose • Center of gravity • 3D acceleration	• Fixed threshold analysis	• Identification of the reason for fall by analyzing blood pressure, heart rate and blood glucose level • Classification of falls by analyzing walking, jumping and fall data from accelerometer
van Schooten et al. (33)	169 older adults (65–99 years) • 109 non-fallers • 60 fallers	• 1 3D accelerometer (trunk-at the level of L5)	• Gait parameters: gait speed, cadence, stride length, and harmonic ratio • Daily activities: number of strides, locomotion duration	• Logistic regression	• 1 week of accelerometry data for each subject was obtained • Locomotion duration and gait variability were significantly correlated with fall history • Predictive ability based on questionnaires, grip strength, trail making test, and gait amount and quality were highly associated with falls
Howcroft et al. (34)	100 older adults (75.5 ± 6.7 years) • 76 non-fallers • 24 fallers	• 2 multipoint pressure sensing insoles (plantar aspect of foot) • 4 3D accelerometer (head, pelvis, and left and right shanks)	• Center of pressure • Impulse variables from ground reaction force • Temporal gait features • Frequency domain features • Ratio of even to odd harmonics (REOH) • Limits of stability (LOS) • Maximum Lyapunov exponent (MLE)	• Multi-layer perceptron neural network (NN) • Naïve Bayes (NB) • Support vector machine (SVM)	• SVM and NN, both provided high accuracy for fall risk classification • NN presented the best performance accuracy (84%) • Single-task gait assessment models performed better than models based on dual-task gait or clinical assessment data
Wang et al. (36)	81 older adults (83.8 ± 3.83 years) • 70 non-multiple fallers • 11 multiple fallers	• 2 3D accelerometer–Opal (center of the lower back and right ankle)	• Gait parameters: cadence, gait variability, and movement vigor • Physiological parameters: – Visual contrast sensitivity – Proprioception – Quadriceps muscle extension strength – Reaction time – Postural sway path	• Partial Spearman correlation	• Normal gait, stair ascent and descent were studied • Gait parameters from stair descent showed higher correlation with physiological profile assessment (PPA) factors than gait parameters from flat surfaces and stair ascent
Brodie et al. (35)	96 older adults (75.5 ± 7.8 years) • 63 non-fallers • 33 fallers	• 1 3D accelerometer and 1 barometer (worn as a pendant)	• Gait parameters: steps per day, cadence, gait variability, gait endurance, and walking adaptability • Physiological parameters: – Visual contrast sensitivity – Proprioception – Quadriceps strength – Reaction time – Postural sway	• Analysis of variance (ANOVA) • Analysis of covariance (ANCOVA)	• Daily-life walking was analyzed • Presented correlation between gait and other factors such as aging, BMI, medications, disability, concern about falling, poor executive function, and higher physiological fall risk
Qiu et al. (37)	100 community-dwelling Korean older women (≥65 years) • 114 non-fallers • 82 fallers	• 5 Xsens inertial sensors−3D Accelerometer + 3D Gyroscope (pelvis, thighs, and shanks)	• Time and frequency domain features from sensory integration test (SIT) • Limits of Stability (LOS) • Sit-to-Stand Five Times (STS5) • Gait parameter from timed up and go (TUG) test: gait velocity, step time and length, tuning time • Range of motion (ROM): knee flexion; knee extension • Choice reaction test (CRT) • Computerized falls efficacy scale (FES)	• Logistic regression • Naïve Bayes (NB) • Decision tree (DT) • Random forest (RF) • Boosted tree (BT) • Support vector machine (SVM)	• Support vector machine for faller classification achieved the highest overall accuracy of 89.4% with 92.7% sensitivity and 84.9% specificity • fallers exhibited worse performances of visual and vestibular systems, a smaller knee ROM, a slower information processing speed, a higher fear of falling, and experienced more difficulties during the complex tasks such as TUG, LOS, and STS5
Rivolta et al. (38)	90 older adults (69.3 ± 16.8 years) • 33 with high risk of falling	• 1 3D accelerometer–GENEActiv (chest)	• Temporal and spatial gait parameters • Balance variables • Tinetti score evaluated from Tinetti test (from 9 balance and 8 gait features)	• Linear regression • Linear model (LM) • Artificial neural network (ANN)	• A large number of features (21) were used to classify the subjects with fall risk • A Tinetti score was used as gold standard • Low misclassification error for ANN (0.11)
Saadeh et al. (39)	20 older adults (65–70 years)	• 1 3D accelerometer–MPU-6050 (upper thigh)	• Fall prediction parameters: – Acceleration–mean and standard deviation (x- and z-axes) – Coefficient of variance–COV (z-axis) – Correlation coefficient between x- and z-axes – Mean amplitude deviation–MAD (x-axis) – Total sum vector–SV • Fall detection parameters: – Total sum vector square–SVS	• Non-linear support vector machine (NLSVM)	• The proposed system included two operation modes: 1) fast mode for fall predication–FMFP (300–700 ms) and 2) slow mode for fall detection-SMFD (within 1 s) • The proposed algorithm accuracy is validated using MobiFall dataset (77 subjects) • Exhibited high sensitivity and specificity for both FMFP (97.8 and 99.1%) and SMFD (98.6 and 99.3%)
Buisseret et al. (40)	73 older adults (83.1 ± 8.3 years) • 50 non-fallers • 23 fallers	• 1 LSM9DS1 inertial sensors−3D Accelerometer + 3D Gyroscope (L4 vertebra)	• Linear acceleration and angular velocity (x-, y- and z-axes) • Parameter from timed up and go (TUG) test: TUG time and gait variability	• Decision tree (DT)	• TUG test results coupled to gait variability parameters presented improved (from 68 to 76%) accuracy of fall risk prediction

### Existing Inertial-Based Sensor Systems for Fall Risk Assessment and Fall Detection

In (31), a piezoresistive accelerometer was used to determine the fall risk. The sensor was placed at the sacrum. The study included TUG (timed-get-up-and-go) and Tinetti tests and measured different gait and functional parameters to assess the fall risk among different subject groups (young control, old control, and older adults with increased fall risk). Analysis of variance (ANOVA) and logistic regression were used to analyze the data and only gait speed and variability showed discriminative result for increased fall risk. Another similar accelerometer-based fall risk assessment study (trunk-at the level of L5) was presented in (33). They also used logistic regression to analyze gait parameters and daily activities and presented good association with falls.

Some researchers suggested other body locations to position the accelerometer sensor such as chest (38) and upper thigh (39) to perform the fall risk assessment and prediction. In (38), the Tinetti score was evaluated from 9 balance and 8 gait features and different machine learning models (linear regression, linear model, and artificial neural network–ANN) were applied to perform fall risk classification. Based on their data, ANN performed the best with a lowest misclassification error of 0.11. Researchers in another article (39) used non-linear support vector machine (NLSVM) for fall prediction and detection operation and achieved high performance sensitivity and specificity for both cases (Table 1).

Two accelerometer-based fall risk assessment study is presented in (36). In this study, the sensors were placed at the center of the lower back and right ankle, and the researchers measured different gait and physiological parameters during normal gait, stair ascent and descent. They used partial Spearman correlation and found a high correlation between gait parameters from stair descent and physiological profile assessment (PPA) factors for fall risk assessment.

Gyroscope measurements along with the accelerometer data were used in two other studies (37, 40). In (40), one inertial sensor unit was positioned at the lower back, at the lumbar 4 vertebra, to measure 3D linear acceleration, angular velocity, and other gait parameters in the TUG test. They used the decision tree algorithm for fall risk prediction. Measurements from five inertial sensors (pelvis, thighs, and shanks) were exploited in (37) to differentiate fallers from non-fallers among a community-dwelling older population. They explored different machine learning algorithms: logistic regression, Naïve Bayes, decision tree, random forest (RF), boosted tree, and support vector machine (SVM) to make a comparison analysis and obtained the highest faller classification accuracy (89.4%) using SVM.

There are other studies (32–35) that incorporated other wearable sensors along with the inertial sensors to explore more relevant features for fall risk assessment. In (35), the researchers used a 3D accelerometer and a barometer worn as a pendant to measure gait and physiological parameters such as steps per day, cadence, gait variability, gait endurance, walking adaptability, visual contrast sensitivity, proprioception, quadriceps strength, reaction time, and postural sway. They used analysis of variance (ANOVA) and analysis of covariance (ANCOVA) to analyze daily life walking and presented a correlation between gait and other factors such as aging, BMI, medications, disability, concern about falling, poor executive function, and higher physiological fall risk. Another study (32) presented a novel fall detection algorithm based on three different sensors: pressure sensor (arm), infra-red sensor (fingertip) and accelerometer (waist). They analyzed blood pressure, heart rate, blood glucose, center of gravity, and 3D acceleration to identify and classify the reasons for fall. Another multi-sensor system that combined two multipoint pressure sensing insoles and four 3D accelerometer (head, pelvis, and left and right shanks) was presented in (34). They applied three machine learning algorithms: multi-layer perceptron neural network (NN), Naïve Bayes (NB), and support vector machine (SVM) to analyze different extracted gait and plantar pressure-related parameters (Table 1). Although both SVM and NN provided high accuracy for fall risk classification, NN presented the best performance accuracy of 84%. The study also observed that single-task gait assessment models performed better than models based on dual-task gait or clinical assessment data.

## Insole-Based Sensor Systems

### Overview

Fall risk assessment and fall detection have been investigated using insole-based sensor systems. Insole-based sensor systems used for gait analysis typically provide measurements regarding plantar pressure distribution and other gait characteristics from the sole of the foot. These systems often use piezoresistive force sensitive resistors (FSRs) embedded in an insole to measure PPD at various foot regions. These pressure sensors have a reduction in resistance as a greater force is applied. The pressure values among all FSRs in an insole can be used to determine the pressure distribution across the foot, which is useful for gaining insight into stance phase gait characteristics (such as stance phase gait events) as well as relative pressures across the plantar aspect of the foot. Some insole-based systems incorporate inertial measurement units (such as accelerometers and gyroscopes) at the plantar aspect of the foot or lower leg to obtain additional gait characteristics. The main components of an insole-based sensor system include the insole base, pressure sensing elements, data acquisition and transmission system, and power source. The data acquired from the wearable insole is usually transmitted to a device for analysis. Upon signal post-processing and data analysis, gait characteristics can be presented in a useful format for patients or clinicians, fall risk can be determined, and meaningful information can be extracted from the investigated parameters.

In addition to using sensors embedded in an insole system to monitor gait characteristics and assess fall risk, physical characteristics of the insole can contribute to reducing fall risk and preventing falls. For example, (53) outline that insoles can allow for balance preservation through controlling the center of pressure of the foot, shock absorption using less rigid materials beneath the hindfoot, providing support at the ankles and stimulating skin receptors to increase reaction speed. Insoles can also vary in elevation to allow for a greater area of the plantar aspect of the foot to be in contact with the sole (54) which can further decrease fall risk.

**Figure 4:**
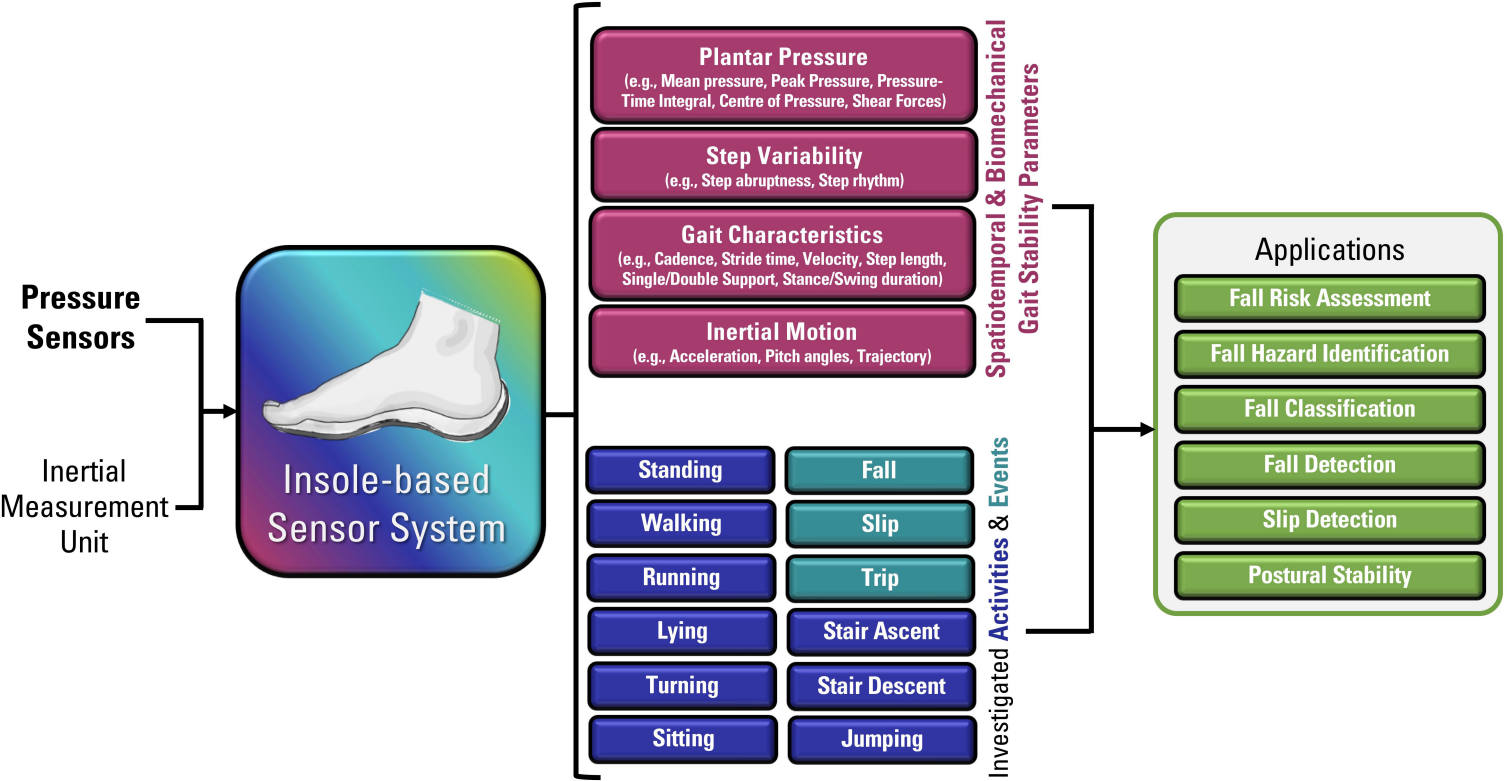
Parameters of interest for fall risk assessment and fall detection using insole-based systems.

Although insole-based systems traditionally mainly focus on foot pressure through plantar pressure distribution measurements, the plantar pressure data can provide meaningful insight into an individual's gait and fall risk. This information when paired with inertial measurements can provide further insight into an individual's fall risk and gait. In fact, fallers and stumblers have shown to exert different pressure values and PPD than non-fallers (55, 56). By recognizing characteristic PPD and gait patterns while walking and standing, further insight into an individual's fall risk can be obtained. The parameters typically analyzed for fall risk assessment or fall detection in existing insole systems (57–67) are shown in Figure 4.

### Existing Insole-Based Systems for Fall Risk Assessment and Fall Detection

In (60), an insole-based system including 13 capacitive pressure sensors and an IMU was developed to assess fall risk in construction workers. This system investigated parameters relevant to biomechanical gait stability through a wireless system. Loss of balance events were studied as they are reported to provide biomechanical parameters regarding gait stability, specifically in the context of external fall risk factors in occupational environments. Changes in those biomechanical parameters were also reported to provide gait metrics regarding safety in workplace environments. The developed insole system was used to simulate loss of balance events in a laboratory setting in addition to assessing normal gait, using data from insoles from both feet. Plantar pressure patterns were used to calculate five parameters relevant to biomechanical gait stability. The equations used by Antwi-Afari and Li (60) to calculate the mean pressure, peak pressure, the pressure-time integral, anterior/posterior center of pressure and medial/lateral center of pressure are presented in Table 2.

**Table 2:** Key points pertaining to insole-based systems used for fall risk assessment and fall detection.

**Reference**	**Sensor system and focus of work**	**Sensor type(s) and number**	**Sensor placement**	**Population**	**Parameters investigated**	**Data acquisition and analysis information**	**Other characteristics**
Kraus et al. (67)	• Insole-based • Physical frailty prediction	Pressure sensors and 6-axis IMU	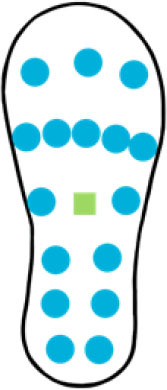	• Orthogeriatric patients • *n* = 57 • 93% women • Mean age: 77 years (SD = 6)	• Number of steps • Stride length • Gait speed • Acceleration over gait cycle • Gait cycle time • Cadence • COP variability • Double support time	• Physical frailty classified using: – SPPB (Short Physical Performance battery) – kNN (k-Nearest Neighbor) – RF (Random Forest)	• *Moticon Science3* insole used
Ayena et al. (63)	• Insole-based with UWB (Ultra-wideband) Radar • Fall risk assessment	Piezoresistive pressure sensors (FSRs) + 3D Accelerometer + Radar system 5 sensors: • 4 FSRs (2 at hindfoot (medial heel and lateral heel), 1 at first metatarsal, and 1 at fifth metatarsal) • 1 3D Accelerometer (outside of sole)	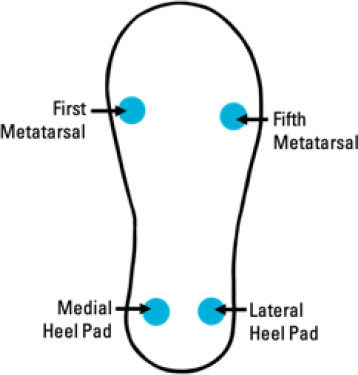	• One healthy young adult participant	• Instrumented insole provides: – Acceleration-related information – Other gait information (Temporal features) – Cadence – Stride time – Stride length – Stride speed • Radar UWB provides information regarding: – Position-based activities (Spatial features) – Stride length – Stride speed	• Risk of Falling Score informed by stride data • Fall risk based on analyzed variability • Fall detection (based on static and dynamic acceleration) • Kalman Filter for Gait Velocity estimation • Algorithm to segment TUG (Timed Up and Go) radar signal: used for stride length, stride time, cadence, stride speed	• FSR diameter: 13 mm • High resolution acceleration measurement: 13-bit and up to ± 16 g • Radar range: 10 m • Radar accuracy: ±10 cm • Low-power radar system
Bucinskas et al. (64)	• Insole-based • Fall Risk Assessment	3 piezoelectric pressure sensors • Pressure sensors developed by researchers using PVC, Velostat and aluminum foil	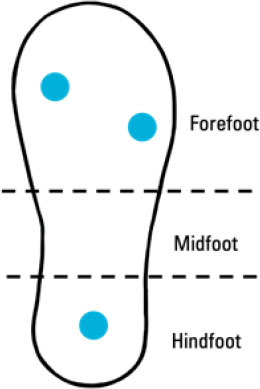	• One participant (three trials)	• Pressure distribution • Duration of stance phases for both feet • Variation of stepping abruptness • Stepping unevenness parameters • Stepping rhythm • Size of step • Gait phase timing	• Analysis of sensor signals in time domain • Correlation-regression analysis for absolute measurement error • Single amplitude values extracted from raw data for load distributions	• Wireless (2.4 GHz WiFi) • Battery-powered 1,300 mAh lithium polymer battery • Activities investigated: “Turnaround,” “Scrolling,” “Upstairs,” “Downstairs,” “Upstairs one by one,” “Walk with left straight leg”
Chen et al. (65)	• Insole-based • Fall Hazard Identification	Pressure sensor array layer (96 pressure sensors)	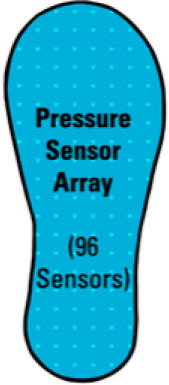	• Healthy individuals • *n* = 10	• Ground reaction force differences • Swing phase acceleration magnitude signal threshold crossing points • Pitch angle at initial foot contact • Pitch angle during midstance • Double support %	• Five features used to train SVM model for fall hazard identification and safe floor activities • One-feature accuracy: 39.54% • Five-feature accuracy: 95.78%	• Device for fall hazard identification • Activities investigated: Walking, Running, Stair ascent, Stair descent
Ji et al. (66)	• Insole-based • Fall Detection	4 FSR pressure sensors (2 at forefoot, 1 at midfoot, 1 at hindfoot)	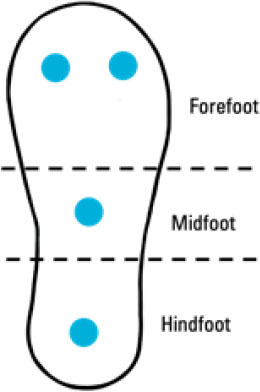	–	• Plantar pressure • Plantar pressure variation • Walking state	–	• Bluetooth data transmission
Antwi-Afari et al. (60)	• Insole-based • Fall Risk Assessment	• 13 Capacitive Pressure sensors: 2 at Toes; 3 at Metatarsal Head; 4 at Arch; 4 at Heel • 1 3D Acceleration sensor at middle of Arch	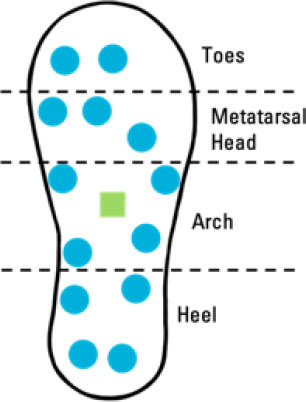	• Construction workers • *n* = 10 • Mean age: 26.50 (SD = 3.35) years	Biomechanical gait stability parameters: • Mean pressure • Peak pressure • Pressure-time integral • Anterior/Posterior center of pressure • Medial/Lateral center of pressure (Investigated through simulation of loss-of-balance events and normal gait)	• 50 Hz pressure sampling rate • Equations used for biomechanical gait stability parameters: • Mean Pressure = $\frac{1}{N}\overset{N}{\underset{i=1}{\sum }}Pi$ • Peak Pressure = Maximum (Pi, …, *PN*) • Pressure-Time Integral = $\overset{N}{\underset{t=1}{\sum }}Pi\times t$ • Anterior/Posterior Center of Pressure = $\frac{\overset{N}{\underset{i=1}{\sum }}XiPi}{\overset{N}{\underset{i=1}{\sum }}Pi}$ • Medial/Lateral Center of Pressure = $\frac{\overset{N}{\underset{i=1}{\sum }}YiPi}{\overset{N}{\underset{i=1}{\sum }}Pi}$ *N* = number of pressure sensors *i* = pressure sensor value (i^th^ sensor) *Xi* and *Yi* = pressure sensor value coordinates	• Wireless Data Transmission • Insole thickness: 2.5 mm • 16 MB flash memory integrated in sole • Pressure range: 0 to 40 N/cm^2^ • Simulated loss-of-balance events = Slip, Trip, Unexpected step-down, Twisted ankle
Cates et al. (61)	• Insole-based • Fall Classification	• 4 Pressure sensors (FSRs): 2 at forefoot and 2 at hindfoot • 1 IMU Sensor at midfoot	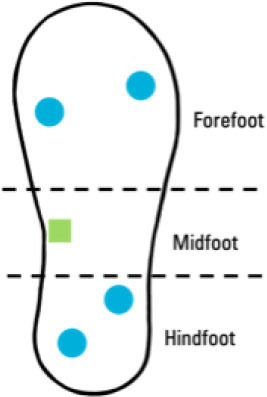	• Healthy males • *n* = 20 • Age: 28 ± 5 years	Low-acceleration Activities of Daily Life (ADL): • Standing • Lying • Sitting • Walking • Running High-acceleration ADLs: • Stair ascent • Stair descent • Jump falls	• Threshold and machine learning methods • Signal of sum vector magnitude filtered using 1^st^ order low-pass butterworth filter (1Hz cut-off) • Support vector machine (SVM) fall detection algorithm • 45 features used for fall classification model • Feature selection using genetic algorithm process • 18 features associated with highest performance of fall detection	• Device for fall classification • 20 Hz sampling rate
Hu et al. (62)	• Insole-based • COP Trajectory	12 FSRs (at toes, metatarsophalangeal joints, foot arch, heel)	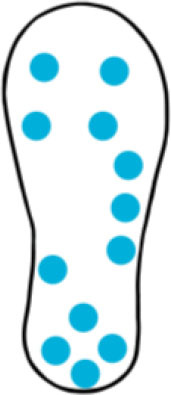	• *n* = 20 • Younger participants: – *n* = 10 – Age: 22.6 ± 1.5 years • Older participants: – *n* = 10 • Age: 65.7 ± 3.4 years	Center of Pressure (COP) Trajectories (indicative of postural control) • Anterior-posterior direction trajectory • Medial-lateral direction trajectory	Non-linear model used to estimate COP more accurately than typical weighted models	• 50 Hz sampling frequency • FSR diameter: 12.7 mm • Bluetooth data transmission
di Rosa et al. (59)	• Insole-based • Fall Risk Score	Pressure sensors + 6D Accelerometer and Gyroscope (Sensors embedded in two layers: Pressure array layer on upper pressure-sensing layer; Other components (including inertial sensors) in second/matrix layer)	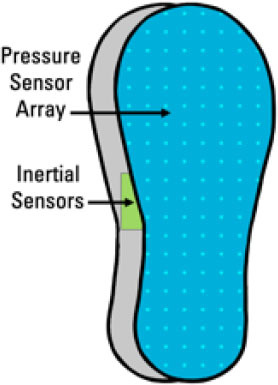	• Older adults (over 65 years) • *n* = 29 • Diverse sample used (sex, health status, mobility, etc.)	• Double support right (fall risk index weighting: 52%) • Single support left (weighting: 31%) • Mediolateral average acceleration amplitude (weighting: 12%) • Heel strike force slope left (weighting: 5%)	• Cluster analysis – Selected indicators: – POMA (Performance Oriented Mobility Assessment Tool) – DGI (Dynamic Gait index) – TUG (Timed Up and Go test)	• Short range communication (Bluetooth) to mobile device • Long range communication from mobile device to computer • Worn during daily activities for 2 weeks • Comprehensive daily activity monitoring not possible (device designed for steady-state gait parameters only)
Das and Kumar (58)	• Insole-based • Postural Stability and Gait Parameters	7 Piezoresistive pressure sensors • Hallux • Metatarsal 1 • Metatarsal 2 • Metatarsal 4 • Metatarsal 5 • Medial Heel • Lateral Heel	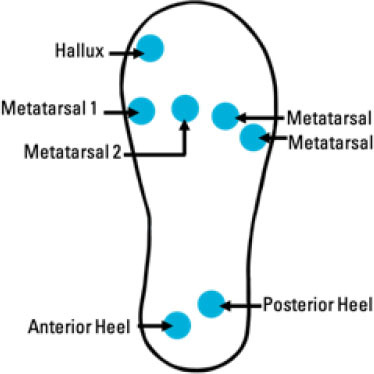	• Healthy males • *n* = 3 • Age range: 22–28 years	Postural stability and spatiotemporal gait parameters: • Plantar Pressure • Force variation during standing and from accidental falls • Gait cycle duration • Stance duration • Swing phase time • Single support	Data filtered using 3^rd^ order Butterworth low-pass filter (cut-ff frequency = 50 Hz)	• Parameters calculated using Heel strike, Heel off, Toe off, Toe strike, Timer • Timer is triggered upon detection of specific gait events • Force range: 0–100 N • Accuracy: ± 2 N
Lincoln and Bamberg (57)	• Insole-based system + camera-based system • Slip Detection	• 6 pressure sensors (FSRs): 4 at forefoot and 2 at heel • 1 3-axis accelerometer (not in sole)	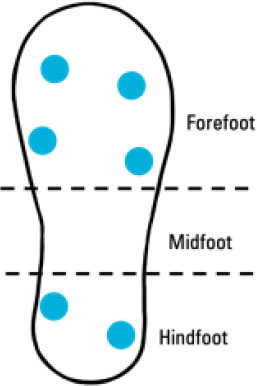	• *n* = 2 • 1 male (age = 23 years) • 1 female (age = 35 years)	Plantar force during slip gait • Normal force • Lateral shear force • Progressional shear force • Body weight acceleration during slip gait	Pressure and acceleration data filtered through low-pass butterworth filter (cut-off frequency = 60 Hz)	• Real-time slip detection • Slips following heel strike were investigated without falls recorded • 114 Hz sampling rate • 90% accuracy

A combined system incorporating an insole with pressure sensors, and acceleration sensor as well as a UWB (ultra-wide band) radar was developed for fall risk assessment by Ayena et al. (63). This system combined information acquired from the insole system and radar system to provide a Risk of Falling score largely based on stride data. A benefit of this system is the non-contact radar system which can be used to assess gait characteristics and fall risk when a wearable device is not necessarily used. When the insole is paired with the radar system, meaningful information such as temporal and spatial gait features can be obtained. The low number force sensitive resistors also reduces the power required for the insole to operate. Various techniques were also used for data analysis which included gait velocity estimation involving the use of a Kalman filter as well as developing an algorithm to segment parameters investigated.

In (64), an insole system was developed for fall risk assessment. The researchers created their own pressure sensor which was made of a 20 x 20 mm sheet of Velostat sandwiched by two 100 μm thick aluminum foil sheets, which is in turn sandwiched by 150 μm thick PVC adhesive film. The sensors in this insole were attached to the lining of the sole, which increases comfort to the user as there is no direct contact of the foot with the piezoelectric sensors. Through wireless transmission, the data acquired from the insole system is transmitted to a microcontroller that is connected to a personal computer. The computer then is used to extract signal amplitude and frequency, which are then used to detect changes in gait and ultimately generate reports and warmings regarding fall diagnosis and gait characteristics. In this work, one participant engaged in six different gait activities (“Turnaround,” “Scrolling,” “Upstairs,” “Downstairs,” “Upstairs one by one,” “Walk with left straight leg”) with three trials per gait activity. The eighteen graphs of data were analyzed to determine parameters such as load distribution, variation in step abruptness and timing of gait phases. This work aimed to develop a system that can be used to detect uncoordinated or unstable gait which would otherwise lead to an increased fall risk.

An insole-based sensor system by Cates et al. (61) was developed to classify falls. This system includes both force sensitive resistors for pressure and an accelerometer from an inertial measurement unit. In all fall detection algorithms used which are IMU-based, the “Sum Vector Magnitude” was calculated, which considers acceleration in x y and z planes. The equation used in this work is:


$$\begin{array}{l} Sum\;Vector\;Magnitude=\sqrt{Ax^{2}+Ay^{2}+Az^{2}} \end{array}$$

where *Ax*^2^ is the acceleration in the *x* direction, *Ay*^2^ is the acceleration in the *y* direction and *Az*^2^is the acceleration in the *z* direction. This method does not depend on the orientation of the sensor, which is beneficial with regards to overcoming any errors caused by the misalignment of the IMU. The Support Vector Machine (SVM) Machine Learning model was used for the fall classification. This model allows for the various low-acceleration activities, high acceleration activities and falls to be distinguished. It is reported that by using both the FSR and inertial data, errors are reduced, specifically with respect to improving the rate of false negatives. Among the 45 features from both the IMU and pressure sensors, 18 were determined to be associated with the highest performance of fall detection. Fourteen of these features are accelerometer-derived and four are derived from pressure sensors. The names of the four FSR-selected features are “*FSR4 switch on duration*,” “*FSR3 total on-off switches*,” “*Mean_FSR3*,” and “*Mean_FSR1 over window's final 2 s*.” The names of the remaining accelerometer-selected features are “*mean_Z*,” “*variance_X*,” “*variance_*Z,” “*variance_Total*,” “*skewness_X*,” “*skewness_Z*,” “*skewness_Total*,” “*correlation_Z*,” “*Minimum filtered Sum Vector Magnitude*,” “*Maximum filtered Sum Vector Magnitude*,” “*filtered Sum Vector Magnitude*<*0.9 duration*” and “*Variance filtered Sum Vector Magnitude over window's final 2 s*” (61).

The work by Chen et al. (65) aimed to address hazard that could lead to falls, with an emphasis on falls in occupational settings. The developed insole consists of four layers: a top fabric layer for comfort, a pressure sensor array (consisting of 96 pressure sensors per foot), an insole-shaped package layer, and the bottom layer consisting of the circuit board and battery. The focus of this work was to identify fall hazards that would result in slips and trips using an insole-based sensor system. This system can be used not only for gait analysis of the user but also to determine whether the floors are safe or are hazardous. To determine if floors were safe or hazardous, activities of daily living including walking, running, stair ascent and stair descent were investigated. The changes in pressure and gait characteristics were also investigated on a slippery surface as individuals adapt their gait to the floor surface. Detecting these characteristic patterns upon negotiation with respect to slippery surfaces and trip negotiation can lead to the identification of hazardous floors which would otherwise lead to falls. A Support Vector Machine model was used in this work and the mean accuracy of a five-fold cross validation test was 95.78%.

Das and Kumar (58) developed an insole-based system to investigate postural stability and gait parameters. This system consists of seven piezoresistive pressure sensors and measurements were in accordance with Zebris mat validation. Parameters pertaining to postural stability and spatiotemporal gait characteristics were determined using a timer associated with specific gait events such as “Heel strike,” “Heel off,” “Toe off,” and “Toe strike.” The millisecond timer is triggered by specific gait events to determine the duration of particular gait events. Like several other insole-based systems, the data acquired from this system is focused on stance phase gait characteristics, with information regarding the swing phase being restricted to the duration of the swing phase.

The “WIISEL” (Wireless Insole for Independent and Safe Elderly Living) system developed by di Rosa et al. (59) consists of sensors embedded in two layers. The top layer consists of an array of pressure sensors and the bottom matrix layer includes other components such as inertial sensors (accelerometer and gyroscope), antenna, Bluetooth protocol and an inductive charging area. This insole was worn by older adults for 2 weeks daily with data acquired from daily activities. This system was designed to obtain information regarding steady-state gait parameters and is thus not able to be used for comprehensive daily activity monitoring. Nonetheless, this system uses the steady state gait characteristics toward a fall risk score. The parameter of greatest contribution to the fall risk score is “Double Support Right” (52% weighting), followed by “Single support left” (31%), “Mediolateral average acceleration amplitude” (12%) and “Heel strike force slope left” (5%). The color-coded fall risk score is displayed on a graphical user interface, where a score of 0–30 is indicative of a low fall risk, 31–70 is indicative of medium fall risk and 71–100 indicates a high fall risk. Due to the complexity of fall risk assessment, a cluster analysis was also completed to ensure the fall risk index can efficiently indicate an individual's risk of falling.

The system developed by Hu et al. (62) was designed to estimate the trajectory of foot center of pressure (COP). COP trajectory and more specifically the COP sway can provide information regarding an individual's fall risk. This work employed a non-linear data analysis model specific to the user to estimate the COP trajectory. It is reported that existing COP trajectory analyses are based on weighted mean approaches, which may not be accurate for all individuals due to intra-individual variability as well as the low number and small sizes of sensors used in this insole. The non-linear user-specific model developed for COP trajectory aims to overcome those limitations.

The system developed by Ji et al. (66) uses four pressure sensors in an insole system designed to detect falls and trigger an alert. This system has two sensors placed at the forefoot, and one at the centers of the midfoot and hindfoot each. By assessing plantar pressure distribution, the variation in the pressure exerted, the walking state or speed of the individual as well as if there is a rapid response form the user, the occurrence of a fall can be determined. Upon detecting a fall, an alert is generated. The user, if conscious, also has the option to press a button upon a fall to generate an alert using this system.

Kraus et al. (67) used the *Moticon Science3* insole to investigate physical frailty. Along with pressure sensors, this insole system consists of a 6-axis inertial measurement unit at the midfoot. This worked aimed to assess the comparability of the data obtained from the insole system with the standard questionnaire for sarcopenia and the TUG (Timed Up and Go test) on predicting physical frailty.

Bipedal slip as investigated by Lincoln and Bamberg (57). An insole-based system consisting of pressure sensors and an IMU attached to the lower leg was used alongside a marker-based motion capture system and floor force plates to investigate the detection of bipedal slips in real time. This system determined the trajectory of plantar forces, acceleration and corresponding readings from the motion capture system to identify the slip motion. The duration difference of a normal step vs. a slip was also determined to be 0.20 and 1.25 s, respectively. While slipping, there is prolonged motion in the progressive direction with a heelstrike occurring thereafter. Slip motions were induced using a low-friction surface of two layers of plastic sheets. In this work where slips were induced through a set-up, only the slip motion was investigated, without investigations regarding falling.

A summary of key points regarding the reviewed insole-based systems used for fall risk assessment and fall detection are presented in Table 2. The placement of pressure sensors in existing systems are depicted as blue circles, with inertial sensors integrated in the insole depicted as green squares. The diagrams to depict the approximate sensor placement locations in the insole are based on figures, images or descriptions included in existing literature, where provided.

## Other Wearable Systems

There are several other wearable sensors that are also investigated for fall risk assessment, fall prevention, and fall detection. It was observed that when a fall event occurred or before the fall event, different physiological parameters such as heart rate, body temperature, sweat rate, lung volume, blood oxygen level, and eye movement of an individual varies from normal (68, 69). Therefore, researchers are also investigating different wearable physiological sensors. For example, wearable photoplethysmography (PPG) and electrocardiography (ECG) sensors are used for blood volume variations and heart rate monitoring (70–73), temperature sensors for body temperature, and electrodermal activity-EDA sensor (also known as galvanic skin response-GSR) to measure skin perspiration and humidity (74). Spirometer is another diagnostic tool that can measure lung capacity and airflow (75). Hence, it can be used for shortness of breath detection due to critical medical conditions and prevent unwanted falls. Moreover, SpO2 (saturation of peripheral oxygen) sensors can monitor blood's oxygen saturation level and electrooculography (EOG) sensors can quantify eye movements by measuring voltage difference between the cornea and retina (75, 76). Some researchers also incorporated global positioning system (GPS) to show the location of the event after detection a fall (77, 78).

## Fall Risk Assessment and Modeling Techniques

In wearable sensor-based fall risk analysis, relevant features are extracted from the sensors data. As the data acquired from the sensors may contain noise and unnecessary information, different filtering techniques are applied to minimize noise and eliminate undesired segment of the data (79). After filtering the data, optimal feature set should be selected for effective analysis. The commonly used features extracted from the wearable sensors are spatiotemporal gait parameters, gait variability, trajectory, center of mass, activity duration and transitions, postural stability, and planter pressure distribution (41, 80). In addition to these extracted features, clinical histories, demographic characteristics, and previous fall records are also important for developing an efficient fall risk assessment model. The approaches used for fall risk assessment can be divided into three categories: Conventional machine learning techniques, Deep learning algorithms, and Knowledge-Driven Model.

Machine learning is a technique that enables the machine (computers/smart devices) to utilize a dataset and to obtain meaningful information that can create a learning model. It uses training data to predict/differentiate a problem based on the data features and generates optimal assessments (76). Deep learning is also a type of machine learning and artificial intelligence (AI). A neural network with multiple hidden layers is recognized as a deep learning method. Deep learning, unlike conventional machine learning algorithms, does not need feature engineering. It is capable to train a large amount of data with high prediction accuracy. However, the hidden layers of a deep learning method are similar to a black box and not easy to understand (81, 82). On the other hand, a knowledge driven model is developed based on evidence, guidelines, and experts' opinions, instead of using observational data (80).

### Conventional Machine Learning Techniques

Machine learning techniques are the most used analyzing approaches for wearable sensor-based fall risk assessment. Conventional machine learning techniques for fall risk modeling and assessment can be divided into two approaches: discriminative and generative. Discriminative techniques are used to generate a decision boundary to classify the subjects into corresponding classes (e.g., fallers and non-fallers). Different discriminative approaches were applied for fall risk assessment such as linear regression (83), logistic regression (84), and Support Vector Machine–SVM (85). On the other hand, generative techniques are used to generate the boundary line of each class instead of having a single decision boundary. Generative models that were reported for fall risk assessment are Naïve Bayes model (86, 87), k-Nearest Neighbor–KNN (86, 88), and Dynamic Bayesian Network–DBN (89).

### Deep Learning

Deep learning is the common name of multi-layer artificial neural networks–ANN. A multi-layer neural network includes three layers: input layer (data features), hidden layer (processing units), and output layer (classes). There are different deep learning techniques that are used for fall risk assessment such as convolutional neural networks–CNN (81, 82, 90), and recurrent neural networks–RNN (82).

### Knowledge-Driven Model

Both machine learning and deep learning models are data-driven approaches. There is another approach that is known as knowledge-driven model and it is based on probabilistic rule and assumptions. The Farseeing fall risk assessment tool (FRAT-up) is such kind of a model which determines the fall risk based on the risk factors related to falls (91). It stores the characteristics of a user in terms of risk factors and based on that knowledge it generates an estimation of the fall risk of the user. However, this model is not fully applicable for wearable sensor-based fall risk assessment as it does not learn from the data.

**Figure 5:**
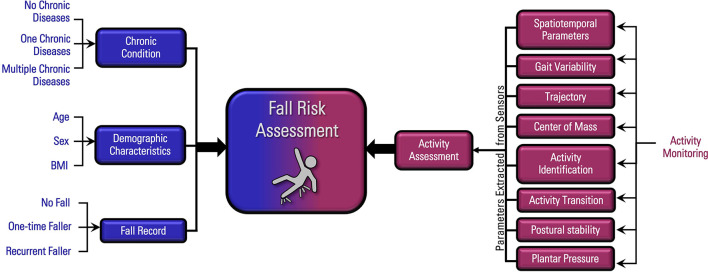
Overall flow diagram of fall risk assessment.

The overall flow diagram of fall risk assessment based on the wearable sensor data, medical history, fall records and demographic characteristics are presented below in Figure 5.

## Challenges

Although major advances were made in the field of wearable sensors in recent years, there are some challenges and limitations of current work that must be overcome.

### Fully Integrated System

Although wearable systems are able to acquire, transmit and present data, further improvements in fully integrated wearable systems can have great benefits to health monitoring. For example, integrating all sensors in a single device, such as pressure and inertial measurement units placed at the plantar aspect of the foot in a single sole, can simplify the wearable system hardware. Improvements to the types of sensors used for more comprehensive monitoring, improving data acquisition, data transmission and power consumption can allow for continuous and long-term monitoring, which can be used to seamlessly monitor an individual's fall risk during daily activities.

### Data Acquisition and Data Transmission

Most studies reviewed obtained pressure readings at a sampling rate of 50 Hz which is sufficient for walking (10); however a higher sampling rate is required for other activities. For example, a che sampling rate is required for activities such as running or cycling, stair ascent and stair descent (92). Although falls commonly occur during activities such as turning or walking on uneven surfaces, activities which require a higher sampling rate, such as running, fast walking, or stair use, are also associated with higher fall risk (36, 93). However, this may increase the power consumption of the device and reduce its operation time. As such, mitigating the trade-off between power consumption and accuracy remains a challenge. Another challenge using wearable devices for daily activity gait analysis is the data transmission range. Most existing devices employ Bluetooth technology, which means that the device receiving data must be in close proximity. Creating a lightweight and small-size data acquisition and storage system that does not impact gait and allows for the device to be used beyond a short range from a mobile device or a computer is a challenge that if overcome, can be very beneficial for gait analysis using wearable devices. Real-time data transmission can also allow for real-time monitoring of the acquired data. Improvements in data analysis and feedback techniques can improve real-time health monitoring using wearable devices.

### Standardization and Consistency

Regarding fall risk assessment, the parameters used vary greatly. A lack of standardization of parameters may make clinical determinations and comparisons from the data acquired from wearable systems more difficult. Regarding insole-based systems, there is great variability in terms of sensor size, number, and placement, which may also lead to difficulties in monitoring and comparing clinically relevant parameters, as variations among devices result in diverse foot regions being investigated in PPD analyses. Therefore, research should be done with a standard protocol and a proper data management to maintain the consistency among fall-related studies.

### Insufficient Sample Size

In order to develop an efficient classification or prediction model for falls, a database with a large number of participants is required. However, due to the requirement of continuous data collection and a long follow-up period, collecting data from a large number of participants becomes time-consuming and costly. As a result, most of the studies had small sample sizes (mostly < 100 participants). This small sample size may result in overfitting of the final classification model. Moreover, insufficient positive samples in comparison to the total sample size may lead to distorted models.

### Generalizability

Since most devices reviewed have tested the accuracy of risk assessment of fall detection in a small sample, it is unclear if the results are generalizable. There is a need to test developed devices on larger sample sizes to ensure the device can be used by the general population. Moreover, using a large sample size that is diverse with regards to age, sex and fall history, can allow for devices to be used by a greater number of individuals. Designing devices that can be used by individuals of varying health conditions as well as individuals with different shoe sizes or limb proportions, can be beneficial and is currently an area in need of improvement.

### Quality of Data

The majority of data presented in the fall studies are generated through experiments where falls are usually simulated in controlled environments. As a result, these data may not accurately reflect real-life conditions. Moreover, real datasets related to fall patterns of older adults with a standardized protocol are not readily available, which is a limitation in proceeding further with fall risk assessment research. Therefore, developing a daily-life continuous wearable monitoring system with a user-friendly design is important to ensure the quality of data for fall-related research.

### User Considerations

Although user considerations regarding comfort, size and flexibility of wearable systems have taken high priority in existing works, there is room for further improvement. Several existing smart insoles are not customizable to the shoe size of the user, and as such are validated using individuals of the same shoe size. Ensuring that insoles are customizable, such as by using materials that are trimmable, can ensure the sole fits appropriately for the user. This is important as an incorrect sole size may result in the sensors not being placed under the anticipated foot regions. Ensuring all wiring is covered and sufficient padding exists for a comfortable device is a challenge, as padding can affect the force distribution in the case of insoles. One benefit of using small and low-cost sensors is the affordability that is translated to the user. Through advances in wearable technologies, smart insoles and inertial sensors for fall risk can be produced at larger quantities to further reduce the end cost to the user. Regarding both IMUs and insole-based systems, it is important for the system to be easy to use, such as by having systems in a plug and play format. Developing graphical user interfaces that are simple enough for the user, while improving biofeedback for the user to make behavioral changes to reduce fall risk are also areas in need of further research.

**Figure 6:**
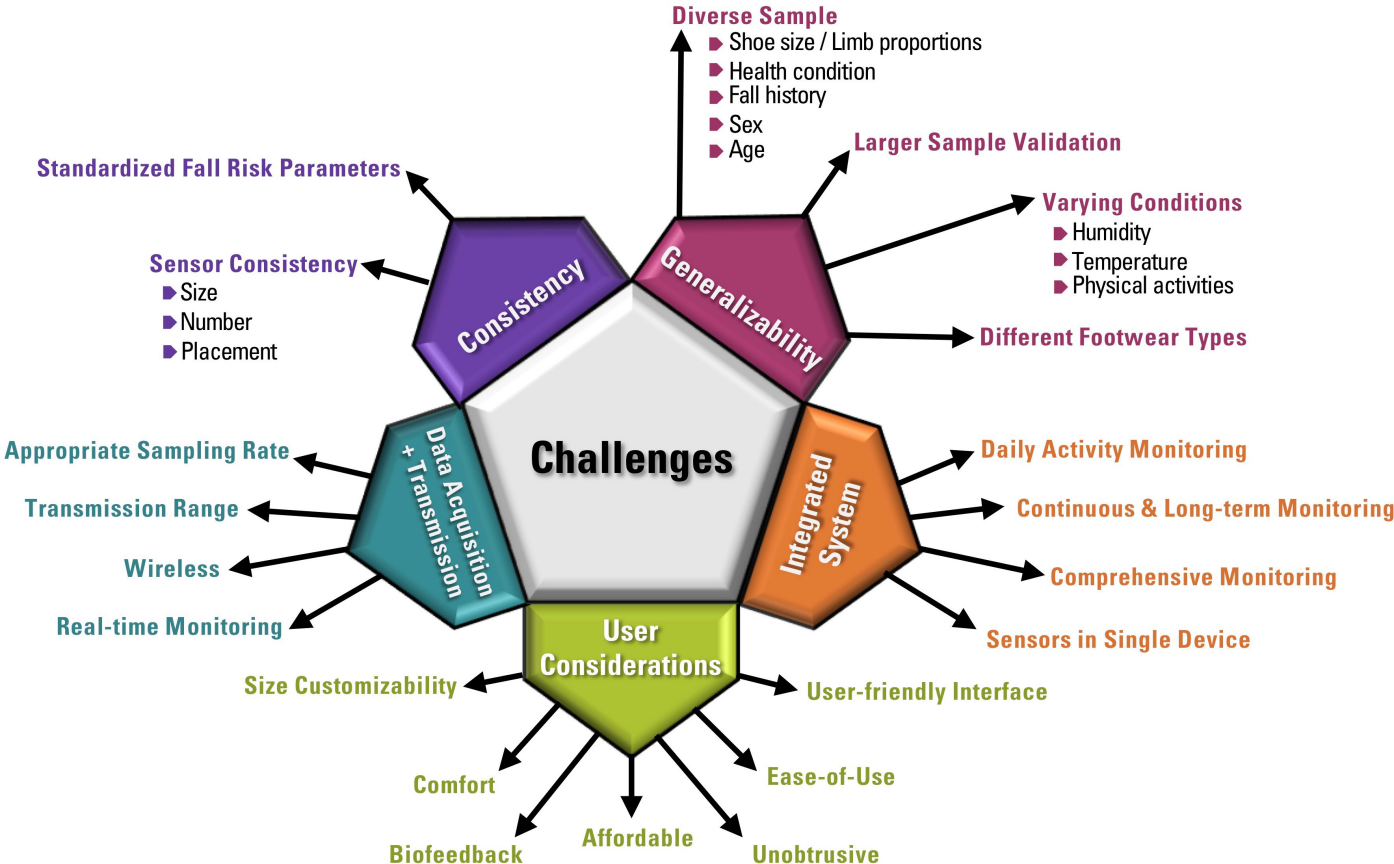
Research challenges associated with fall risk assessment using wearable insole-based and inertial-based devices.

Research challenges associated with fall risk assessment with wearable insole-based and inertial-based devices are presented below in Figure 6.

## Future Research Perspectives

Additional research in the field of wearable sensors for fall risk assessment and fall detection can allow for more meaningful advances in the field to be made. One area of future work can include the strategic combination of the reviewed technologies. For instance, incorporating several diverse sensor types, such as PPG, EMG, temperature, pressure and inertial sensors in a single device can lead to a more comprehensive activity monitoring device (10). Employing sensor fusion techniques may also lead to meaningful associations among diverse parameters. Standardizing the placement of sensors and the parameters investigated in future works can allow for more meaningful and clinically relevant comparisons and analyses to be made.

As an individual's fall risk can be affected by several factors such as age, sex, ethnicity, medical conditions, determining which parameters are most relevant for specific populations can lead to more efficient analyses of fall risk in diverse populations. Therefore, constructing a public database with a standardized protocol could help in comparing the results from a new system and reuse the data for further in-depth analysis.

The hardware and computational resources for a fall risk assessment system can be a crucial factor for continuous and long-term communication and monitoring. Therefore, high-performance hardware support is required with an efficient algorithm to handle large and complicated data efficiently. On the other hand, high-performance hardware typically requires more power which is also one of the most critical factors to be considered while developing a wearable system. Therefore, to build a balanced system, the power requirement of the system should be minimized by utilizing advanced power-efficient electronics and power sources. Energy harvesting can be an alternative to solve this issue.

Finally, incorporating personalized biofeedback in a user-friendly manner can be useful in future systems, as users would be able to make corrective behavioral changes to reduce their risk of falling based on feedback from their analyzed activities. With the integration of internet of things (IoT) with wearable sensor technology, it would be possible to implement this personalized biofeedback fall application. It would enable the execution of advanced learning algorithms to effectively analyze complicated fall-related data in real time. The edge, fog, and cloud layers of an IoT architecture would be able to provide processing, storage, data management, and decision (prediction, prevention, and decision) for fall cases. In addition, incorporation of deep reinforcement learning will expand assessment alternatives with respect to different environments, while preserving robustness, accuracy and data privacy.

## Conclusions

In recent years, fall risk assessment is gaining prominence with the realization that falls require significant medical attention and can pose huge financial and social burdens. Development of an effective low-cost, user-friendly, wearable sensor-based fall risk assessment tool combined with advanced wireless communications and machine learning algorithms can significantly advance fall-related studies (clinical and non-clinical). In this article, the recent and the most popular wearable technologies developed for fall risk assessment and fall detection were reviewed. Therefore, we mainly emphasized different proposed inertial sensor-based and insole-based systems and did a comprehensive survey as these two are the most common and reliable wearable systems for fall-related studies. While performing this survey, we also took into account the publications that mainly focused on real-time monitoring. Although the focus of this article is on inertial sensor-based and insole-based systems which examine gait characteristics to assess fall risk, other wearable technologies were also reviewed. In addition, we included a brief review on different fall risk assessment and modeling techniques in order to provide an overview of a complete wearable fall risk assessment system. By addressing the key points of existing technologies, challenges to overcome as well as future research perspectives, it is expected that the information in this article can be used to gain a thorough understanding of existing wearable sensor technologies and to improve future wearable devices developed for fall risk assessment.

## Author Contributions

Research idea, critical revisions, supervision, and funding: MJD. Research plan and review and editing: MJD, SS, and AF. Original draft preparation and figure creation: SS and AF. Database search: SS. All authors contributed to the article and approved the submitted version.

## Funding

This work was supported in part by the Canada Research Chair program and the Natural Sciences and Engineering Research Council (NSERC) of Canada.

## Conflict of Interest

The authors declare that the research was conducted in the absence of any commercial or financial relationships that could be construed as a potential conflict of interest.

## Publisher's Note

All claims expressed in this article are solely those of the authors and do not necessarily represent those of their affiliated organizations, or those of the publisher, the editors and the reviewers. Any product that may be evaluated in this article, or claim that may be made by its manufacturer, is not guaranteed or endorsed by the publisher.

## References

[1] Majumder S Mondal T Deen M. Wearable sensors for remote health monitoring. Sensors. (2017) 17:130. 10.3390/s17010130 28085085 PMC5298703

[2] Deen MJ. Information and communications technologies for elderly ubiquitous healthcare in a smart home. Pers Ubiquitous Comput. (2015) 19:573–99. 10.1007/s00779-015-0856-x

[3] Nisar K Ibrahim Ag A Ag Wu L Adamov A Deen MJ. Smart home for elderly living using wireless sensor networks and an android application. In: 2016 IEEE 10th International Conference on Application of Information and Communication Technologies (AICT) (London: IEEE) (2016). p. 1–8. 10.1109/ICAICT.2016.7991655

[4] Majumder S Aghayi E Noferesti M Memarzadeh-Tehran H Mondal T Pang Z. Smart homes for elderly healthcare—recent advances and research challenges. Sensors. (2017) 17:2496. 10.3390/s17112496 29088123 PMC5712846

[5] Faisal AI Majumder S Mondal T Cowan D Naseh S Deen MJ. Monitoring methods of human body joints: state-of-the-art and research challenges. Sensors. (2019) 19:2629. 10.3390/s19112629 31185629 PMC6603670

[6] Majumder S Deen MJ. Smartphone sensors for health monitoring and diagnosis. Sensors. (2019) 19:2164. 10.3390/s19092164 31075985 PMC6539461

[7] Wu Q Zhang YD Tao W Amin MG. Radar-based fall detection based on Doppler time–frequency signatures for assisted living. IET Radar Sonar Navigat. (2015) 9:164–72. 10.1049/iet-rsn.2014.0250

[8] Diraco G Leone A Siciliano P. A radar-based smart sensor for unobtrusive elderly monitoring in ambient assisted living applications. Biosensors. (2017) 7:55. 10.3390/bios7040055 29186786 PMC5746778

[9] Islam SMM Boric-Lubecke O Lubekce VM. Concurrent respiration monitoring of multiple subjects by phase-comparison monopulse radar using independent component analysis (ICA) with JADE algorithm and direction of arrival (DOA). IEEE Access. (2020) 8:73558–69. 10.1109/ACCESS.2020.2988038

[10] Subramaniam S Majumder S Faisal AI Deen MJ. Insole-based systems for health monitoring: current solutions and research challenges. Sensors. (2022) 22:438. 10.3390/s22020438 35062398 PMC8780030

[11] Faisal AI Majumder S Scott R Mondal T Cowan D Deen MJ. A simple low-cost multi-sensor-based smart wearable knee monitoring system. IEEE Sens J. (2021) 21:8253–66. 10.1109/JSEN.2020.3044784

[12] Jiang W Majumder S Kumar S Subramaniam S Li X Khedri R. A wearable tele-health system towards monitoring COVID-19 and chronic diseases. IEEE Rev Biomed Eng. (2022) 15:61–84. 10.1109/RBME.2021.3069815 33784625 PMC8905615

[13] Faisal AI Mondal T Cowan D Deen MJ. Characterization of knee and gait features from a wearable tele-health monitoring system. IEEE Sens J. (2022) 22:4741–53. 10.1109/JSEN.2022.3146617

[14] Bonato P. Advances in wearable technology and applications in physical medicine and rehabilitation. J Neuroeng Rehabil. (2005) 2:2. 10.1186/1743-0003-2-2 15733322 PMC552335

[15] Tao W Liu T Zheng R Feng H. Gait analysis using wearable sensors. Sensors. (2012) 12:2255–83. 10.3390/s120202255 22438763 PMC3304165

[16] Muro-de-la-Herran A Garcia-Zapirain B Mendez-Zorrilla A. Gait analysis methods: an overview of wearable and non-wearable systems, highlighting clinical applications. Sensors. (2014) 14:3362–94. 10.3390/s140203362 24556672 PMC3958266

[17] Khow KSF Visvanathan R. Falls in the aging population. Clin Geriatr Med. (2017) 33:357–68. 10.1016/j.cger.2017.03.002 28689568

[18] Howland J Peterson EW Levin WC Fried L Pordon D Bak S. Fear of falling among the community-dwelling elderly. J Aging Health. (1993) 5:229–43. 10.1177/089826439300500205 10125446

[19] Statistics Canada. Understanding Seniors' Risk of Falling and Their Perception of Risk (2015). Available online at: https://www150.statcan.gc.ca/n1/pub/82-624-x/2014001/article/14010-eng.htm (accessed March 26, 2022).

[20] Distefano G Goodpaster BH. Effects of exercise and aging on skeletal muscle. Cold Spring Harb Perspect Med. (2018) 8:a029785. 10.1101/cshperspect.a029785 28432116 PMC5830901

[21] Perell KL Nelson A Goldman RL Luther SL Prieto-Lewis N Rubenstein LZ. Fall risk assessment measures: an analytic review. J Gerontol A Biol Sci Med Sci. (2001) 56:M761–6. 10.1093/gerona/56.12.M761 11723150

[22] John Hopkins Medicine. John Hopkins Fall Risk Assessment Tool for Home Health Care (2007). Available online at: https://www.hopkinsmedicine.org/institute_nursing/_docs/JHFRAT/Home%20Health%20with%20Watermark.PNG (accessed March 29, 2022).

[23] McCarthy M. Falls are leading cause of injury deaths among older people, US study finds. BMJ. (2016) 354:i5190. 10.1136/bmj.i5190

[24] Palumbo P Palmerini L Bandinelli S Chiari L. Fall risk assessment tools for elderly living in the community: can we do better? PLoS ONE. (2015) 10:e0146247. 10.1371/journal.pone.0146247 26716861 PMC4696849

[25] Favre J Jolles BM Aissaoui R Aminian K. Ambulatory measurement of 3D knee joint angle. J Biomech. (2008) 41:1029–35. 10.1016/j.jbiomech.2007.12.003 18222459

[26] Bakhshi S Mahoor MH Davidson BS. Development of a body joint angle measurement system using IMU sensors. In 2011 Annual International Conference of the IEEE Engineering in Medicine and Biology Society. (Boston, MA: IEEE) (2011). p. 6923–6. 10.1109/IEMBS.2011.6091743 22255930

[27] Seel T Raisch J Schauer T. IMU-based joint angle measurement for gait analysis. Sensors. (2014) 14:6891–909. 10.3390/s140406891 24743160 PMC4029684

[28] Castañeda JJ Ruiz-Olaya AF Lara-Herrera CN Roldán FZ. Knee Joint Angle Monitoring System Based on Inertial Measurement Units for Human Gait Analysis. (2017). p. 690–3. 10.1007/978-981-10-4086-3_173

[29] Crews DJ. Real-Time Estimation of Knee Angle, Heel-Strike, and Toe-Off Events for Gait Rehabilitation Devices (2017).

[30] Pathirana PN Karunarathne MS Williams GL Nam PT Durrant-Whyte H. Robust and accurate capture of human joint pose using an inertial sensor. IEEE J Transl Eng Health Med. (2018) 6:1–11. 10.1109/JTEHM.2018.2877980 30456000 PMC6237710

[31] Bautmans I Jansen B van Keymolen B Mets T. Reliability and clinical correlates of 3D-accelerometry based gait analysis outcomes according to age and fall-risk. Gait Posture. (2011) 33:366–72. 10.1016/j.gaitpost.2010.12.003 21227697

[32] Kumar SV Manikandan K Kumar N. Novel fall detection algorithm for the elderly people. In 2014 International Conference on Science Engineering and Management Research (ICSEMR) (Chennai: IEEE). (2014). p. 1–3. 10.1109/ICSEMR.2014.7043578

[33] van Schooten KS Pijnappels M Rispens SM Elders PJM Lips P van Dieën JH. Ambulatory fall-risk assessment: amount and quality of daily-life gait predict falls in older adults. J Gerontol A Biol Sci Med Sci. (2015) 70:608–15. 10.1093/gerona/glu225 25568095

[34] Howcroft J Lemaire ED Kofman J. Wearable-sensor-based classification models of faller status in older adults. PLoS ONE. (2016) 11:e0153240. 10.1371/journal.pone.0153240 27054878 PMC4824398

[35] Brodie MA Coppens MJ Ejupi A Gschwind YJ Annegarn J Schoene D. Comparison between clinical gait and daily-life gait assessments of fall risk in older people. Geriatr Gerontol Int. (2017) 17:2274–82. 10.1111/ggi.12979 28176431

[36] Wang K Delbaere K Brodie MAD Lovell NH Kark L Lord SR. Differences between gait on stairs and flat surfaces in relation to fall risk and future falls. IEEE J Biomed Health Inform. (2017) 21:1479–86. 10.1109/JBHI.2017.2677901 28278486

[37] Qiu H Rehman RZU Yu X Xiong S. Application of wearable inertial sensors and a new test battery for distinguishing retrospective fallers from non-fallers among community-dwelling older people. Sci Rep. (2018) 8:16349. 10.1038/s41598-018-34671-6 30397282 PMC6218502

[38] Rivolta MW Aktaruzzaman Md Rizzo G Lafortuna CL Ferrarin M Bovi G. Evaluation of the Tinetti score and fall risk assessment via accelerometry-based movement analysis. Artif Intell Med. (2019) 95:38–47. 10.1016/j.artmed.2018.08.005 30195985

[39] Saadeh W Butt SA Altaf MA. A patient-specific single sensor iot-based wearable fall prediction and detection system. IEEE Trans Neural Syst Rehabil Eng. (2019) 27:995–1003. 10.1109/TNSRE.2019.2911602 30998473

[40] Buisseret F Catinus L Grenard R Jojczyk L Fievez D Barvaux V. Timed up and go and six-minute walking tests with wearable inertial sensor: one step further for the prediction of the risk of fall in elderly nursing home people. Sensors. (2020) 20:3207. 10.3390/s20113207 32516995 PMC7309155

[41] Hemmatpour M Ferrero R Montrucchio B Rebaudengo M. A Review on fall prediction and prevention system for personal devices: evaluation and experimental results. Adv Human Comput Interact. (2019) 2019:1–12. 10.1155/2019/9610567

[42] Guimaraes V Ribeiro D Rosado L. A smartphone-based fall risk assessment tool: measuring one leg standing, sit to stand and falls efficacy scale. In 2013 IEEE 15th International Conference on e-Health Networking, Applications and Services (Healthcom 2013). (Lisbon: IEEE) (2013). p. 529–33. 10.1109/HealthCom.2013.6720733

[43] Begg R Best R Dell'Oro L Taylor S. Minimum foot clearance during walking: Strategies for the minimisation of trip-related falls. Gait Posture. (2007) 25:191–8. 10.1016/j.gaitpost.2006.03.008 16678418

[44] Jiang S Zhang B Wei D. The elderly fall risk assessment and prediction based on gait analysis. In 2011 IEEE 11th International Conference on Computer and Information Technology. (IEEE). (2011). p. 176–80. 10.1109/CIT.2011.82

[45] Shumway-Cook A Brauer S Woollacott M. Predicting the probability for falls in community-dwelling older adults using the timed up andamp; go test. Phys Ther. (2000) 80:896–903. 10.1093/ptj/80.9.896 10960937

[46] Muir SW Berg K Chesworth B Speechley M. Use of the berg balance scale for predicting multiple falls in community-dwelling elderly people: a prospective study. Phys Ther. (2008) 88:449–59. 10.2522/ptj.20070251 18218822

[47] Najafi B Aminian K Loew F Blanc Y Robert PA. Measurement of stand-sit and sit-stand transitions using a miniature gyroscope and its application in fall risk evaluation in the elderly. IEEE Transac Biomed Eng. (2002) 49:843–51. 10.1109/TBME.2002.800763 12148823

[48] Vellas BJ Wayne SJ Romero L Baumgartner RN Rubenstein LZ Garry PJ. One-leg balance is an important predictor of injurious falls in older persons. J Am Geriatr Soc. (1997) 45:735–8. 10.1111/j.1532-5415.1997.tb01479.x 9180669

[49] Tucker CA. Measuring walking. Pediatr Phys Ther. (2014) 26:469. 10.1097/PEP.0000000000000087

[50] Doi T Hirata S Ono R Tsutsumimoto K Misu S Ando H. The harmonic ratio of trunk acceleration predicts falling among older people: results of a 1-year prospective study. J Neuroeng Rehabil. (2013) 10:7. 10.1186/1743-0003-10-7 23356576 PMC3562223

[51] Toebes MJP Hoozemans MJM Furrer R Dekker J van Dieën JH. Local dynamic stability and variability of gait are associated with fall history in elderly subjects. Gait Posture. (2012) 36:527–31. 10.1016/j.gaitpost.2012.05.016 22748312

[52] Riva F Toebes MJP Pijnappels M Stagni R van Dieën JH. Estimating fall risk with inertial sensors using gait stability measures that do not require step detection. Gait Posture. (2013) 38:170–4. 10.1016/j.gaitpost.2013.05.002 23726429

[53] Nagano H Begg R. Shoe-insole technology for injury prevention in walking. Sensors. (2018) 18:1468. 10.3390/s18051468 29738486 PMC5982664

[54] Shanthikumar S Low Z Falvey E McCrory P Franklyn-Miller A. The effect of gait velocity on calcaneal balance at heel strike; Implications for orthotic prescription in injury prevention. Gait Posture. (2010) 31:9–12. 10.1016/j.gaitpost.2009.08.003 19880320

[55] Mickle KJ Munro BJ Lord SR Menz HB Steele JR. Foot pain, plantar pressures, and falls in older people: a prospective study. J Am Geriatr Soc. (2010) 58:1936–40. 10.1111/j.1532-5415.2010.03061.x 20831725

[56] Niu J Zheng Y Liu H Chen X Ran L. Stumbling prediction based on plantar pressure distribution. Work. (2019) 64:705–12. 10.3233/WOR-193032 31815710

[57] Lincoln LS Bamberg SJM. Insole sensor system for real-time detection of biped slip. In 2010 Annual International Conference of the IEEE Engineering in Medicine and Biology. (Buenos Aires: IEEE). (2010). p. 1449–52. 10.1109/IEMBS.2010.5626859 21096354

[58] Das R Kumar N. Investigations on postural stability and spatiotemporal parameters of human gait using developed wearable smart insole. J Med Eng Technol. (2015) 39:75–8. 10.3109/03091902.2014.968676 25350821

[59] di Rosa M Hausdorff JM Stara V Rossi L Glynn L Casey M. Concurrent validation of an index to estimate fall risk in community dwelling seniors through a wireless sensor insole system: a pilot study. Gait Posture. (2017) 55:6–11. 10.1016/j.gaitpost.2017.03.037 28407507

[60] Antwi-Afari MF Li H. Fall risk assessment of construction workers based on biomechanical gait stability parameters using wearable insole pressure system. Adv Eng Inform. (2018) 38:683–94. 10.1016/j.aei.2018.10.002

[61] Cates B Sim T Heo H Kim B Kim H Mun J. A novel detection model and its optimal features to classify falls from low- and high-acceleration activities of daily life using an insole sensor system. Sensors. (2018) 18:1227. 10.3390/s18041227 29673165 PMC5948845

[62] Hu X Zhao J Peng D Sun Z Qu X. Estimation of foot plantar center of pressure trajectories with low-cost instrumented insoles using an individual-specific nonlinear model. Sensors. (2018) 18:421. 10.3390/s18020421 29389857 PMC5855500

[63] Ayena JC Chioukh L Otis MJ-D Deslandes D. Risk of falling in a timed up and go test using an uwb radar and an instrumented insole. Sensors. (2021) 21:722. 10.3390/s21030722 33494509 PMC7866057

[64] Bucinskas V Dzedzickis A Rozene J Subaciute-Zemaitiene J Satkauskas I Uvarovas V. Wearable feet pressure sensor for human gait and falling diagnosis. Sensors. (2021) 21:5240. 10.3390/s21155240 34372477 PMC8347941

[65] Chen D Asaeikheybari G Chen H Xu W Huang M-C. Ubiquitous fall hazard identification with smart insole. IEEE J Biomed Health Inform. (2021) 25:2768–76. 10.1109/JBHI.2020.3046701 33351772

[66] Ji Z He S Zhou Z Ma Y Wu S Chen L. Design of wearable fall detection alarm insole. In 2021 13th International Conference on Measuring Technology and Mechatronics Automation (ICMTMA). (Beihai: IEEE). (2021). p. 42–5. 10.1109/ICMTMA52658.2021.00019

[67] Kraus M Saller MM Baumbach SF Neuerburg C Stumpf UC Böcker W. Prediction of physical frailty in orthogeriatric patients using sensor insole–based gait analysis and machine learning algorithms: cross-sectional study. JMIR Med Inform. (2022) 10:e32724. 10.2196/32724 34989684 PMC8771341

[68] Horta ET Lopes IC Rodrigues JJPC Proenca ML. A mobile health application for falls detection and biofeedback monitoring. In 2013 IEEE 15th International Conference on e-Health Networking, Applications and Services (Healthcom 2013). (Lisbon: IEEE) (2013). p. 36–40. 10.1109/HealthCom.2013.6720634

[69] Bhati N. mHealth based ubiquitous fall detection for elderly people. In 2017 8th International Conference on Computing, Communication and Networking Technologies (ICCCNT) (IEEE) (2017). p. 1–7. 10.1109/ICCCNT.2017.8204033

[70] Nemati E Deen M Mondal T. A wireless wearable ECG sensor for long-term applications. IEEE Commun Mag. (2012) 50:36–43. 10.1109/MCOM.2012.6122530

[71] Zhou C-C Tu C-L Gao Y Wang F-X Gong H-W Lian P. A low-power, wireless, wrist-worn device for long time heart rate monitoring and fall detection. In 2014 International Conference on Orange Technologies. (Xi'an: IEEE). (2014). p. 33–6. 10.1109/ICOT.2014.6954670

[72] Malheiros L Daniel Amvame Nze G Xavier Cardoso L. Fall detection system and Body positioning with heart rate monitoring. IEEE Lat Am Trans. (2017) 15:1021–6. 10.1109/TLA.2017.7932688

[73] Majumder S Chen L Marinov O Chen C-H Mondal T Deen MJ. Noncontact wearable wireless ECG systems for long-term monitoring. IEEE Rev Biomed Eng. (2018) 11:306–21. 10.1109/RBME.2018.2840336 29993585

[74] Bilgin TT Erdogan SZ. A data mining approach for fall detection by using k-nearest neighbour algorithm on wireless sensor network data. IET Communications. (2012) 6:3281–7. 10.1049/iet-com.2011.0228

[75] Karar ME Shehata HI Reyad O. A Survey of IoT-based fall detection for aiding elderly care: sensors, methods, challenges and future trends. Appl Sci. (2022) 12:3276. 10.3390/app12073276

[76] Mozaffari N Rezazadeh J Farahbakhsh R Yazdani S Sandrasegaran K. Practical fall detection based on IoT technologies: a survey. Internet Things. (2019) 8:100124. 10.1016/j.iot.2019.100124

[77] Kau L-J Chen C-S. A Smart phone-based pocket fall accident detection, positioning, and rescue system. IEEE J Biomed Health Inform. (2015) 19:44–56. 10.1109/JBHI.2014.2328593 25486656

[78] Rihana S Mondalak J. Wearable fall detection system. In 2016 3rd Middle East Conference on Biomedical Engineering (MECBME). (Beirut: IEEE). (2016). p. 84–7. 10.1109/MECBME.2016.7745414

[79] Bo J Hoai Thu T Baek E Sakong S Xiao J Mondal T. Walking-age analyzer for healthcare applications. IEEE J Biomed Health Inform. (2014) 18:1034–42. 10.1109/JBHI.2013.2296873 24808231

[80] Zhao G Chen L Ning H. Sensor-based fall risk assessment: a survey. Healthcare. (2021) 9:1448. 10.3390/healthcare9111448 34828494 PMC8624006

[81] Nait Aicha A Englebienne G van Schooten K Pijnappels M Kröse B. Deep learning to predict falls in older adults based on daily-life trunk accelerometry. Sensors. (2018) 18:1654. 10.3390/s18051654 29786659 PMC5981199

[82] Tunca C Salur G Ersoy C. Deep Learning for Fall Risk Assessment with inertial sensors: utilizing domain knowledge in spatio-temporal gait parameters. IEEE J Biomed Health Inform. (2020) 24:1994–2005. 10.1109/JBHI.2019.2958879 31831454

[83] Liu Y Redmond SJ Narayanan MR Lovell NH. Classification between non-multiple fallers and multiple fallers using a triaxial accelerometry-based system. in 2011 Annual International Conference of the IEEE Engineering in Medicine and Biology Society (IEEE) (2011). p. 1499–502. 10.1109/IEMBS.2011.6090342 22254604

[84] Doheny EP Walsh C Foran T Greene BR Fan CW Cunningham C. Falls classification using tri-axial accelerometers during the five-times-sit-to-stand test. Gait Posture. (2013) 38:1021–5. 10.1016/j.gaitpost.2013.05.013 23791781

[85] Greene BR McGrath D Walsh L Doheny EP McKeown D Garattini C. Quantitative falls risk estimation through multi-sensor assessment of standing balance. Physiol Meas. (2012) 33:2049–63. 10.1088/0967-3334/33/12/2049 23151494

[86] Caby B Kieffer S de Saint Hubert M Cremer G Macq B. Feature extraction and selection for objective gait analysis and fall risk assessment by accelerometry. Biomed Eng Online. (2011) 10:1. 10.1186/1475-925X-10-1 21244718 PMC3022766

[87] Colagiorgio P Romano F Sardi F Moraschini M Sozzi A Bejor M. Affordable, automatic quantitative fall risk assessment based on clinical balance scales and Kinect data. In 2014 36th Annual International Conference of the IEEE Engineering in Medicine and Biology Society. (Chicago, IL: IEEE). (2014). p. 3500–3. 10.1109/EMBC.2014.6944377 25570745

[88] Simila H Mantyjarvi J Merilahti J Lindholm M Ermes M. Accelerometry-Based berg balance scale score estimation. IEEE J Biomed Health Inform. (2014) 18:1114–21. 10.1109/JBHI.2013.2288940 24235319

[89] Cuaya G Muñoz-Meléndez A Carrera LN Morales EF Quiñones I Pérez AI. A dynamic bayesian network for estimating the risk of falls from real gait data. Med Biol Eng Comput. (2013) 51:29–37. 10.1007/s11517-012-0960-2 23065654

[90] Savadkoohi M Oladunni T Thompson LA. Deep neural networks for human's fall-risk prediction using force-plate time series signal. Expert Syst Appl. (2021) 182:115220. 10.1016/j.eswa.2021.115220 PMC9540455 36211616

[91] Cattelani L Chesani F Palumbo P Palmerini L Bandinelli S Becker C. FRAT-Up, a Rule-Based System Evaluating Fall Risk in the Elderly. In 2014 IEEE 27th International Symposium on Computer-Based Medical Systems. (New York, NY: IEEE). (2014). p. 38–41. 10.1109/CBMS.2014.35

[92] Abdul Razak AH Zayegh A Begg RK Wahab Y. Foot plantar pressure measurement system: a review. Sensors. (2012) 12:9884–912. 10.3390/s120709884 23012576 PMC3444133

[93] Quach L Galica AM Jones RN Procter-Gray E Manor B Hannan MT. The nonlinear relationship between gait speed and falls: the maintenance of balance, independent living, intellect, and zest in the elderly of boston study. J Am Geriatr Soc. (2011) 59:1069–73. 10.1111/j.1532-5415.2011.03408.x 21649615 PMC3141220

